# High-dose saccharin supplementation does not induce gut microbiota changes or glucose intolerance in healthy humans and mice

**DOI:** 10.1186/s40168-020-00976-w

**Published:** 2021-01-12

**Authors:** Joan Serrano, Kathleen R. Smith, Audra L. Crouch, Vandana Sharma, Fanchao Yi, Veronika Vargova, Traci E. LaMoia, Lydia M. Dupont, Vanida Serna, Fenfen Tang, Laisa Gomes-Dias, Joshua J. Blakeslee, Emmanuel Hatzakis, Scott N. Peterson, Matthew Anderson, Richard E. Pratley, George A. Kyriazis

**Affiliations:** 1 Department of Biological Chemistry & Pharmacology, College of Medicine, The Ohio State University, Columbus, OH USA; 2 Department of Microbiology, College of Arts & Sciences, The Ohio State University, Columbus, OH USA; 3 Sanford Burnham Prebys Medical Discovery Institute, La Jolla, CA USA; 4 Translational Research Institute for Metabolism and Diabetes, Advent-Health, Orlando, FL USA; 5 Department of Food Science and Technology, College of Food, Agricultural & Environmental Sciences, The Ohio State University, Columbus, OH USA; 6 Department of Horticulture and Crop Science, College of Food, Agricultural & Environmental Sciences, The Ohio State University, Columbus, OH USA

**Keywords:** Artificial sweeteners, Saccharin, Sweet taste receptors, Gut microbiota, Glucose intolerance, Short-chain fatty acids, Fecal metabolomics, T1R2, Dysbiosis

## Abstract

**Background:**

Non-caloric artificial sweeteners (NCAS) are widely used as a substitute for dietary sugars to control body weight or glycemia. Paradoxically, some interventional studies in humans and rodents have shown unfavorable changes in glucose homeostasis in response to NCAS consumption. The causative mechanisms are largely unknown, but adverse changes in gut microbiota have been proposed to mediate these effects. These findings have raised concerns about NCAS safety and called into question their broad use, but further physiological and dietary considerations must be first addressed before these results are generalized. We also reasoned that, since NCAS are bona fide ligands for sweet taste receptors (STRs) expressed in the intestine, some metabolic effects associated with NCAS use could be attributed to a common mechanism involving the host.

**Results:**

We conducted a double-blind, placebo-controlled, parallel arm study exploring the effects of pure saccharin compound on gut microbiota and glucose tolerance in healthy men and women. Participants were randomized to placebo, saccharin, lactisole (STR inhibitor), or saccharin with lactisole administered in capsules twice daily to achieve the maximum acceptable daily intake for 2 weeks. In parallel, we performed a 10-week study administering pure saccharin at a high dose in the drinking water of chow-fed mice with genetic ablation of STRs (T1R2-KO) and wild-type (WT) littermate controls. In humans and mice, none of the interventions affected glucose or hormonal responses to an oral glucose tolerance test (OGTT) or glucose absorption in mice. Similarly, pure saccharin supplementation did not alter microbial diversity or composition at any taxonomic level in humans and mice alike. No treatment effects were also noted in readouts of microbial activity such as fecal metabolites or short-chain fatty acids (SCFA). However, compared to WT, T1R2-KO mice were protected from age-dependent increases in fecal SCFA and the development of glucose intolerance.

**Conclusions:**

Short-term saccharin consumption at maximum acceptable levels is not sufficient to alter gut microbiota or induce glucose intolerance in apparently healthy humans and mice.

**Trial registration:**

Trial registration number NCT03032640, registered on January 26, 2017.

Video abstract

**Supplementary Information:**

The online version contains supplementary material available at 10.1186/s40168-020-00976-w.

## Background

Non-caloric artificial sweeteners (NCAS) are often consumed as a substitute for dietary sugars, limiting the caloric content of food without compromising its palatability. Six NCAS are approved as food additives in the USA (saccharin, aspartame, acesulfame potassium (AceK), sucralose, neotame, and advantame) by the Food and Drug Administration (FDA). The use of NCAS has increased dramatically over the past decade [1, 2], due to growing awareness of the negative health outcomes associated with high sugar overconsumption [3]. Strikingly, NCAS use in children has tripled in a decade [4] with recent estimates suggesting that 25% of children and 41% of adults in the USA are daily consumers of NCAS [4].

Paradoxically, although many epidemiological studies do not find any effect of NCAS consumption on the risk of type 2 diabetes mellitus (T2DM) or metabolic syndrome [5–11], several other studies have noted positive associations between NCAS intake and these conditions [12–21]. These findings have raised concerns among consumers and health professionals alike that NCAS may not be physiologically inert, as originally thought, and their general use may lead to adverse metabolic outcomes [22]. However, a large number of interventional studies did not demonstrate significant effects of NCAS consumption on glycemic control [23–36], yet evidence from a few interventional studies support the former viewpoint [37–40]. The pathophysiological mechanisms that possibly cause these adverse associations have been intensely speculated, but remain largely unknown [41]. Interestingly, a number of animal studies have reported effects of NCAS intake on gut microbiota [37, 42–51], but one study in particular established a causative relationship between saccharin consumption and the development of glucose intolerance through adverse changes in gut microbiota [37]. This report, which also included a small number of human participants, revived concerns about the use of NCAS and questioned their safety. However, a recent clinical trial using sucralose did not confirm these outcomes [34]. Furthermore, sucralose supplementation in ileitis-prone SAMP mice caused some changes in gut microbiota, but glucose tolerance was not affected [52].

Collectively, these variable outcomes may reflect differences in the NCAS used, the characteristics of the studied population, and the accompanied diet or other methodological considerations related to these reports. It is thus conceivable that some of these factors can interact or synergize with NCAS to produce differential effects in the measured outcomes. Consequently, the causative relationship between NCAS consumption and adverse metabolic outcomes should be further evaluated after accounting for possible contributions associated with these factors. We also reasoned that since NCAS are bona fide ligands for sweet taste receptors (STRs), some of the effects associated with NCAS use could be attributed to a common mechanism involving the host. Beyond the tongue, STRs are expressed in a variety of tissues including the gastrointestinal tract [53]. Intestinal STRs play a role in regulating metabolic responses to the ingestion of sugars [54, 55], so it is reasonable to speculate that STR-mediated chemosensation in the gastrointestinal tract may provide a mechanistic link between NCAS, gut microbiota, and metabolic regulation.

To explore whether NCAS consumption is an independent modulator of gut microbiota and glucose tolerance and to address the potential involvement of intestinal STRs, we conducted a comprehensive translational investigation involving humans and rodents. First, we performed a randomized, double-blind, placebo-controlled interventional study during which the diet of healthy participants was supplemented for 2 weeks with capsules that contained saccharin at the maximum acceptable daily intake (ADI), lactisole (a human-specific inhibitor of human STRs), saccharin with lactisole, or placebo. Second, to address contributions of STR signaling and to explore potential effects that may require higher saccharin doses and time of exposure, we performed a corresponding 10-week study that exceeded the maximum saccharin ADI in chow-fed mice carrying a genetic ablation of STRs (T1R2-KO) or wild-type controls (WT).

## Results

### Human participants

A total of fifty-four participants were randomized to four treatment groups. Forty-six subjects completed the study and were included in all analyses. Eight participants were excluded from the analysis due to non-compliance (Supp. Figure 1). The clinical characteristics of all participants are summarized in Supp. table 1. At baseline, no differences in basic anthropometric and metabolic parameters were noted between treatment groups (Table 1). All participants complied with the physical activity and dietary requirements of the study (see the “Methods” section) and consumed the expected dose for the treatment period (Supp. table 2). None of the interventions significantly altered body weight (paired *t* test, placebo, *p* = 0.33; saccharin, *p* = 0.21; lactisole, *p* = 0.25; Sac + Lac, *p* = 0.96). No adverse effects of any treatment were reported.

**Table 1:** Baseline characteristics of participants

Total, *n* (M/F)	Placebo	Saccharin	Lactisole	Sac + Lac	*P* value
	11 (3/8)	13 (4/9)	12 (2/10)	10 (5/5)	
Age, year	24.91 ± 1.59	28.91 ± 2.60	32.92 ± 2.78	28.80 ± 2.91	0.199
Height, cm	166.61 ± 2.37	169.03 ± 3.31	164.54 ± 1.92	172.53 ± 2.23	0.494
Weight, kg	59.00 ± 1.81	64.52 ± 3.49	62.13 ± 1.90	66.57 ± 2.64	0.305
BMI, kg/m^2^	21.29 ± 0.62	22.40 ± 0.53	22.93 ± 0.47	22.38 ± 0.78	0.261
Glucose, mg/dL	87.55 ± 2.40	92.00 ± 2.41	91.63 ± 2.64	90.00 ± 1.23	0.519
Triglycerides, mg/dL	72.36 ± 7.93	71.82 ± 13.88	87.42 ± 12.85	65.70 ± 7.57	0.605
Total cholesterol, mg/dL	166.91 ± 9.38	163.82 ± 10.09	182.33 ± 8.25	154.60 ± 7.47	0.333
HDL, mg/dL	66.55 ± 4.12	57.55 ± 3.74	62.58 ± 4.60	64.80 ± 3.96	0.236
LDL, mg/dL	85.91 ± 7.92	91.82 ± 7.47	102.25 ± 7.29	76.50 ± 9.24	0.162
Cholesterol:HDL ratio	2.58 ± 0.18	2.95 ± 0.27	3.08 ± 0.28	2.49 ± 0.20	0.103
LDL:HDL ratio	1.36 ± 0.17	1.67 ± 0.22	1.77 ± 0.25	1.26 ± 0.19	0.115

All values are mean ± SEM. Baseline differences between groups were assessed by ANCOVA using sex as covariate

*M/F* male/female, *BMI* body mass index, *HDL* high-density cholesterol, *LDL* low-density cholesterol, *AUC* area under the curve

### Glucose tolerance and ex vivo intestinal function

**Fig. 1:**
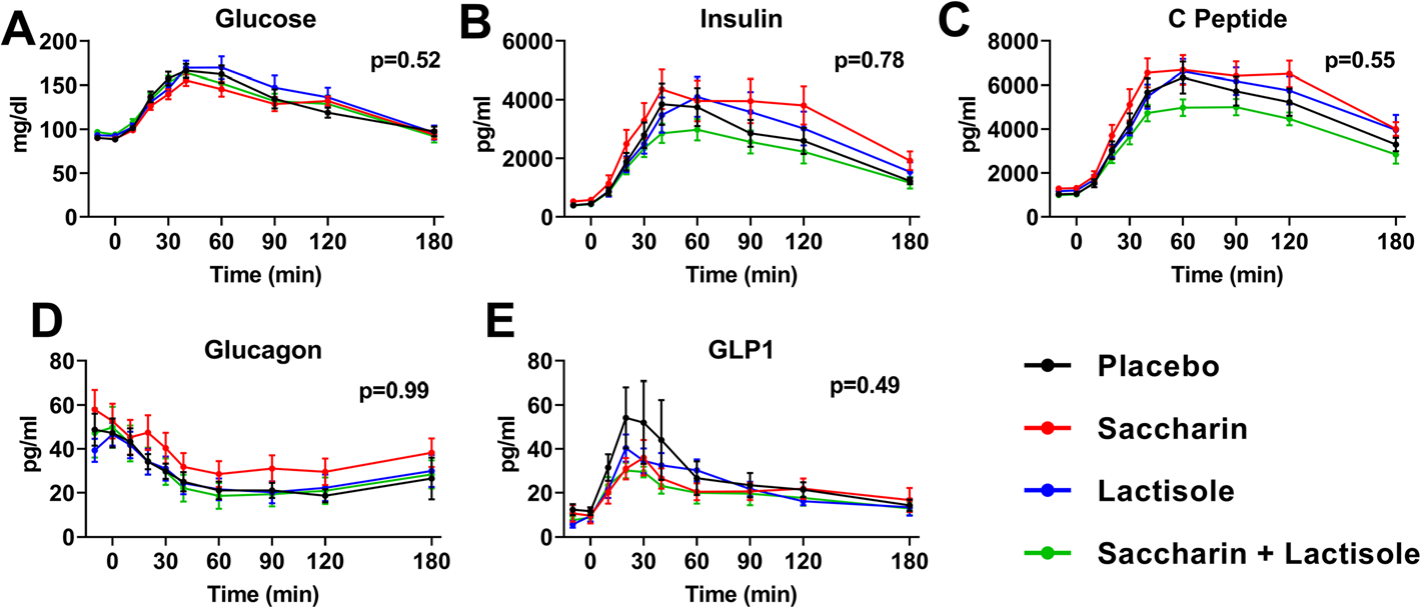
Effects of saccharin and/or lactisole treatment on glucose tolerance in humans. Plasma excursions of **a** glucose, **b** insulin, **c** C-peptide, **d** glucagon, and **e** GLP-1 in response to an oral glucose challenge after 2 weeks of treatment (*n* = 10–13/group). Two-way ANOVA repeated measures, *p* value of time x treatment effect

Two weeks of continuous supplementation of pure saccharin at a dose equal to ADI [56] did not alter glucose responses to a 75-g oral glucose tolerance test (OGTT) among participants (Fig. 1a and Table 2). To test for possible delayed effects of the treatment, we assessed glucose tolerance after a 2-week recovery period during which all groups received placebo. No differences in glucose excursions were observed between the post-treatment and recovery (washout) periods (Supp. figure 2A). Similar to glucose responses, plasma excursions of insulin, C-peptide, glucagon, or glucagon-like peptide 1 (GLP-1) were not different between treatment groups (Fig. 1b–e and Table 2) or within subjects (pre vs. post) of each treatment (Supp. figure 2B-E). The achieved statistical power was 90%.

**Table 2:** Glucose and hormonal excursions in an oral glucose tolerance test after the intervention

	Placebo	Saccharin	Lactisole	Sac + Lac	ANCOVA P
Glucose (AUC)	7136.4 ± 943.6	6606.9 ± 951.7	7571.0 ± 1289.2	6203.8 ± 1084.2	0.6018
Insulin (AUC)	362518.7 ± 55694.9	476124.6 ± 77735.0	412000.5 ± 69663.6	299078.8 ± 41026.4	0.7627
C peptide (AUC)	645605.0 ± 73479.8	747311.4 ± 68855.8	677199.5 ± 61852.4	525798.3 ± 28535.9	0.6034
Glucagon (AUC)	− 3993.5 ± 771.9	− 3226.6 ± 1306.1	− 3768.0 ± 984.4	− 4504.1 ± 1528.9	0.8632
GLP1 (AUC)	2734.8 ± 983.2	2195.4 ± 410.7	2294.1 ± 384.5	1862.9 ± 520.9	0.0662

All values are mean ± SEM. Treatment effects between groups were assessed by ANCOVA using the baseline glucose tolerance test AUC as a covariate

**Fig. 2:**
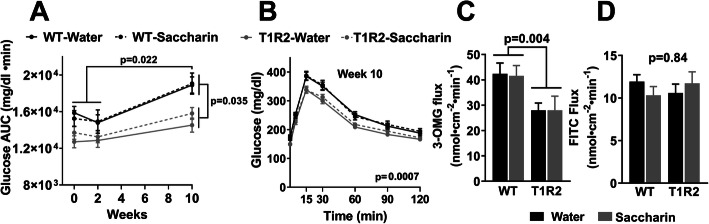
Effects of saccharin treatment on glucose homeostasis in mice. **a** Glucose responses during an i.g.GTT expressed as area under curve (AUC) before 0 week, 2 and 10 weeks after water or saccharin treatment in WT and T1R2-KO (T1R2) mice. Two-way ANOVA main genotype effect, *p* < 0.0001; *p* values of post hoc test. **b** Glucose excursions during an i.g.GTT after 10 weeks of water or saccharin treatment. Two-way ANOVA repeated measures, *p* value of main genotype effect. **c** Ex vivo glucose flux using 3-O-methy-glucose (3-OMG) in intact mouse intestines following 10 weeks of water or saccharin treatment. Two-way ANOVA, *p* value of main genotype effect. **d** Ex vivo intestinal permeability assessed by FITC-dextran (4 kDa) flux in intact mouse intestines following 10 weeks of water or saccharin treatment. Two-way ANOVA, *p* value of main treatment effect. For mouse in vivo studies (*n* = 23–28/group), for ex vivo studies (*n* = 6–11/group)

Next, we addressed the long-term effects of high-dose saccharin supplementation on glucose tolerance in mice and specifically explored the role of NCAS sensing by intestinal STRs. Ad libitum chow-fed WT and T1R2-KO mice were supplemented with pure saccharin in the drinking water for 10 weeks to achieve daily consumption equal to 4 times the human ADI adjusted for mouse body surface area [57]. The actual saccharin consumption was similar to the target consumption for both genotypes (Supp. figure 3A-B), and it did not affect food intake (Supp. figure 3C). Saccharin consumption did not cause differences in body weight gain compared to water alone in either genotype (Supp. figure 3D). As in humans, saccharin treatment had no effect on glucose tolerance (i.e., intra-gastric GTT (IG.GTT)) in WT or T1R2-KO mice assessed after 2 or 10 weeks of treatment (Fig. 2a, b). However, we did observe age-dependent increases in IG.GTT responses of WT mice. Notably, these effects were absent in T1R2-KO mice, which also had reduced IG.GTT responses compared to WT littermates [58] (Fig. 2a).

Although chronic saccharin treatment was unsuccessful in modifying glucose tolerance in mice, it may have induced localized intestinal changes that may contribute to long-term metabolic susceptibility. To address this possibility, we assessed ex vivo glucose transport post-treatment using intact intestines (Ussing chamber) from treated mice and found no effect of saccharin supplementation in the transport of the non-metabolizable glucose analog 3-O-methyl-glucose (3-OMG). However, we observed decreased glucose transport in T1R2-KO intestines, consistent with the IG.GTT data and previous observations [58] (Fig. 2c). In addition, saccharin supplementation did not change the expression of STRs or glucose transporters in either genotype (Supp. figure 3E). Because gut microbiota can alter intestinal permeability [59–61] and saccharin treatment was shown to disrupt epithelial cell barrier in Caco-2 cell monolayers [62], we assessed ex vivo FITC-dextran (4 kDa) flux in treated intact intestines, but found no differences between treatments or genotypes (Fig. 2d).

### Gut microbiota

Saccharin-induced glucose intolerance was previously shown to be contingent upon direct changes in gut microbiota composition [37], so we performed 16S rRNA gene sequencing of fecal samples from the human and mouse studies to investigate whether alterations in microbial communities are induced in response to treatments despite the absence of metabolic responses.

Prior to the interventions (pre-treatment), human participants of all treatment groups had similar gut microbial alpha diversity assessed using the Chao1 index to measure species richness (i.e., observed count values) and the Shannon and Simpson indices to measure taxonomic diversity and evenness [63, 64] (Supp. figure 4A, 5A). Similarly, non-metric multidimensional scaling (NMDS) plot of beta diversity (Bray-Curtis distance matrix) showed no differences between treatment groups prior to the intervention (Supp. figure 4C and 5C). Thus, no baseline differences were noted in the relative microbial abundances between groups at the genus (Supp. figure 4E) or any other taxonomic rank (Supp. table 5). Finally, no gender-dependent baseline differences in alpha (Supp. figure 4B and 5B) or beta diversity (Supp. figure 4D and 5D) were observed.

**Fig. 3:**
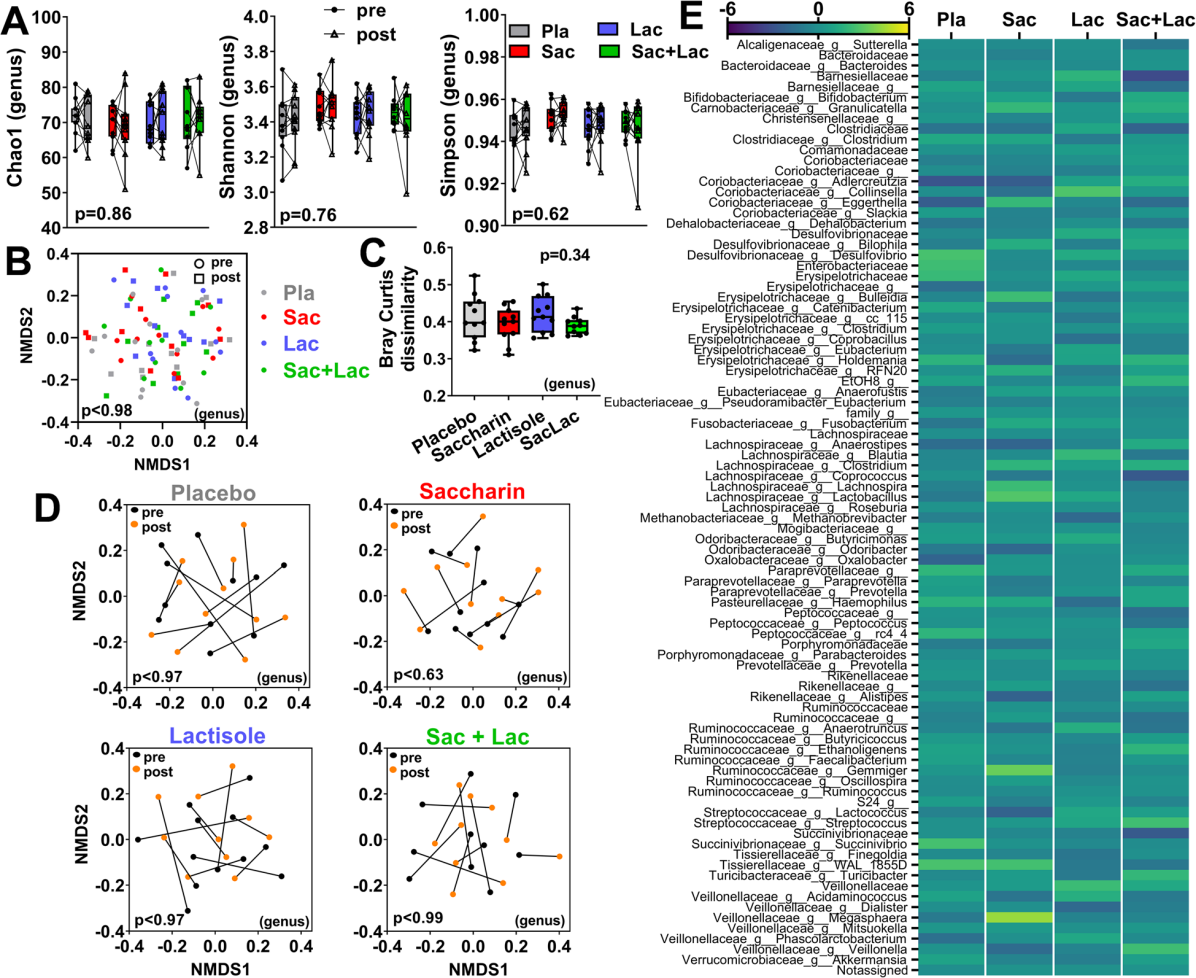
Treatment effects on gut microbial diversity and composition (genus) in humans. **a** Alpha diversity indices (Chao1, Shannon, and Simpson) pre- and post-treatment (lines connect data from the same participant; detailed statistics, Supp. Table.3). **b** Nonmetric multidimensional scaling (NMDS) plots of Bray-Curtis dissimilarities between all groups pre- and post-treatment, or **c** within each treatment group (lines connect data from the same participant). **d** Within-subject Bray-Curtis dissimilarity (paired pre-post) for each treatment group (detailed statistics of beta diversity, Supp. Table.4). e Average values (arbitrary units) of pre-post compositional changes (Δ) at the genus level for each treatment (detailed statistics, Supp. Table.5). For **a**, two-way ANOVA repeated measures, *p* value of time x treatment effect. For **b**, **c**, PERMANOVA *p* value. For **d**, ANOVA *p* value

Next, we assessed the effects between treatments and the within subject responses to treatment in microbial communities. The degree of microbial alpha diversity was not altered in response to any treatment (pre-post) or between treatment groups (Fig. 3a and Supp. figure 5E). NMDS plot of beta diversity showed no clear clustering by treatment (Fig. 3b and Supp. figure 5F). Next, we assessed within subject responses to each treatment, but none of the NMDS plots revealed any significant shift (Fig. 3c and Supp. figure 5G). In agreement, the Bray-Curtis dissimilarity index (pre-post) (Fig. 3d and Supp. figure 5H) or the weighted UniFrac distances (Supp. figure 5I) were similar between treatments. These data suggest there are no major changes in microbial communities in response to any treatment or between treatments. Furthermore, we assessed whether there are specific changes in the relative abundance of individual taxa, but found no significant treatment-effect and responses among treatments were equivalent (Fig. 3e and Supp. table 5). Finally, we implemented a linear discriminant analysis (LDA) effect size (LEfSe) [65] to determine whether potential differential effects for each treatment can be explained by specific taxonomic biomarkers, but the modeling did not produce any differentially regulated features (Supp. table 6).

**Fig. 4:**
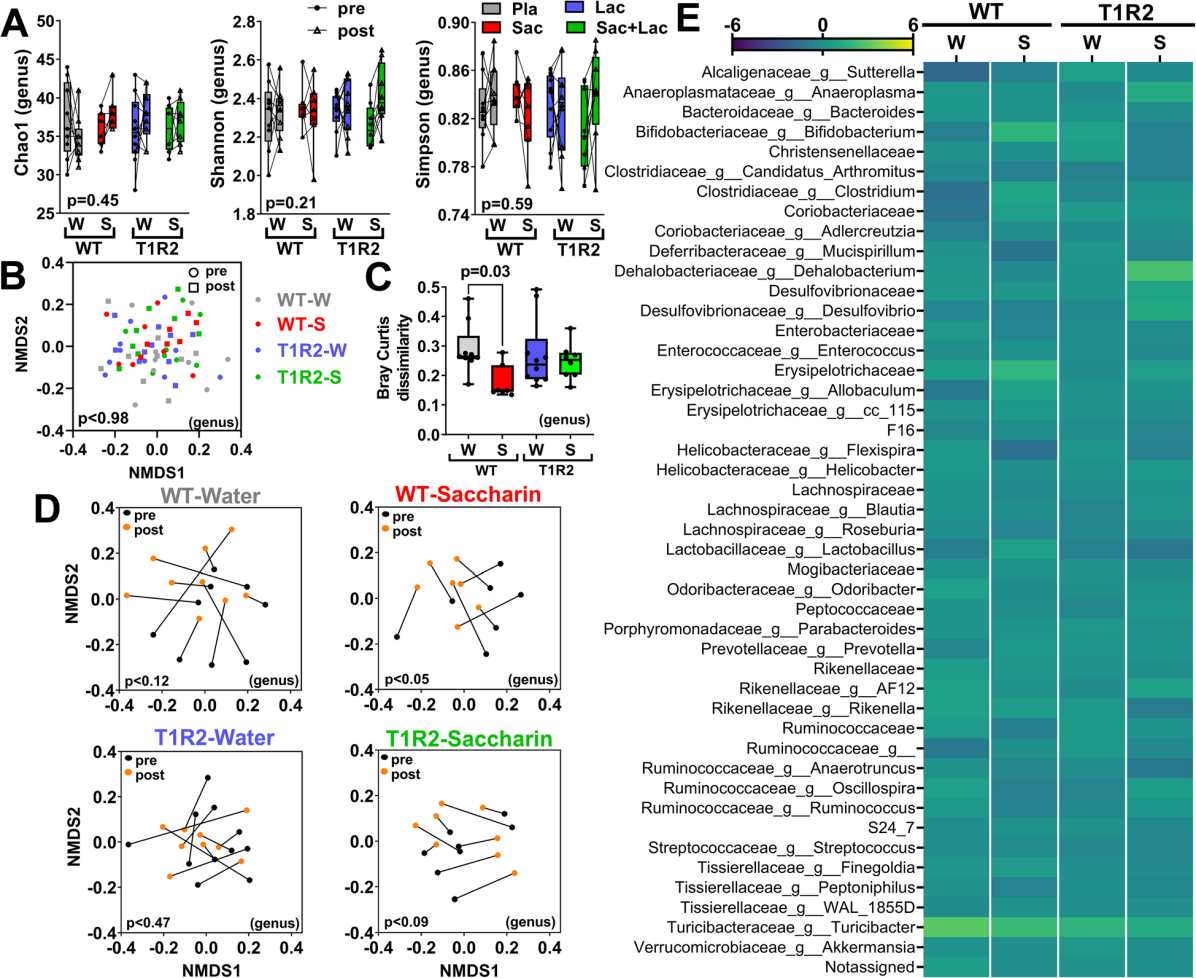
Treatment effects on gut microbial diversity and composition (genus) in mice. **a** Alpha diversity indices (Chao1, Shannon, and Simpson) pre- and post-treatment (lines connect data from the same mouse; detailed statistics, Supp. Table.7). **b** Nonmetric multidimensional scaling (NMDS) plots of Bray-Curtis dissimilarities between all groups pre- and post-treatment, or **c** within each group (lines connect data from the same participant). **d** Within-subject Bray-Curtis dissimilarity (paired pre-post) for each treatment group (detailed statistics of beta diversity, Supp. Table.8). **e** Average values (arbitrary units) of pre-post compositional changes (Δ) at the genus level for each treatment (detailed statistics, Supp. Table.9). For **a**, two-way ANOVA repeated measures, *p* value of time x treatment effect. For **b**, **c**, PERMANOVA *p* value. For **d**, Kruskal-Wallis *p* value. W water, S saccharin

In mice, we did not observe baseline differences among the treatment group or genotypes in alpha diversity indices (Supp. figure 6A-B and 7A-B), in NMDS plots of beta diversity (Supp. figure 6C-D and 7C-D), or in relative microbial abundances (Supp. figure 6E and Suppl. table 9). Despite the larger dose and longer duration of treatment in mice, pure saccharin did not alter alpha diversity indices (Fig. 4a and Supp. figure 7E) or the NMDS plot of beta diversity between treatments (Fig. 4b and Supp. figure 7F). However, when we analyzed within subject responses to each treatment, WT mice fed saccharin showed a moderate, but significant clustering (Fig. 4c and Supp. figure 7G). This trend was also manifested as a lower Bray-Curtis dissimilarity index (Fig. 4d and Supp. figure 7H), suggesting that, compared to other treatments, saccharin feeding in WT mice induced less overall changes in microbial profiles over time (pre- vs. post-treatment). However, consistent with this finding, saccharin supplementation did not cause any changes in the relative abundance of individual taxa (Fig. 4e and Supp. table 9), or the weighted UniFrac distances (Supp. figure 7I), nor it produced any differentially regulated features in LEfSe analysis (Supp. table 10).

Finally, we wanted to eliminate the possibility that the multiple group comparisons of our design had reduced statistical sensitivity, masking a main saccharin treatment effect on gut microbiota. So, in addition to between- and within-group comparisons, we independently performed a complete statistical analysis using pairwise comparisons only between the control (placebo or water) and saccharin treatment groups in human and mouse samples. Pairwise comparisons produced identical statistical outcomes to multiple group comparisons (Supp. table 12). Next, we calculated the magnitude of a potential saccharin effect on the microbiome [66] and found that the mean difference in pairwise comparisons for all metrics was < 0.5 standard deviations. For instance, at the genus level, we calculated a small size effect for the Bray-Curtis index (Cohen’s *d* = 0.38) and a negligible size effect for the Shannon diversity index (Cohen’s *d* = 0.14). Based on these measures, we estimated that the assessment of such subtle effects would require > 93 subjects per group in pairwise comparisons, further confirming the lack of a major effect of saccharin on microbiome.

### Fecal metabolomics

**Fig. 5:**
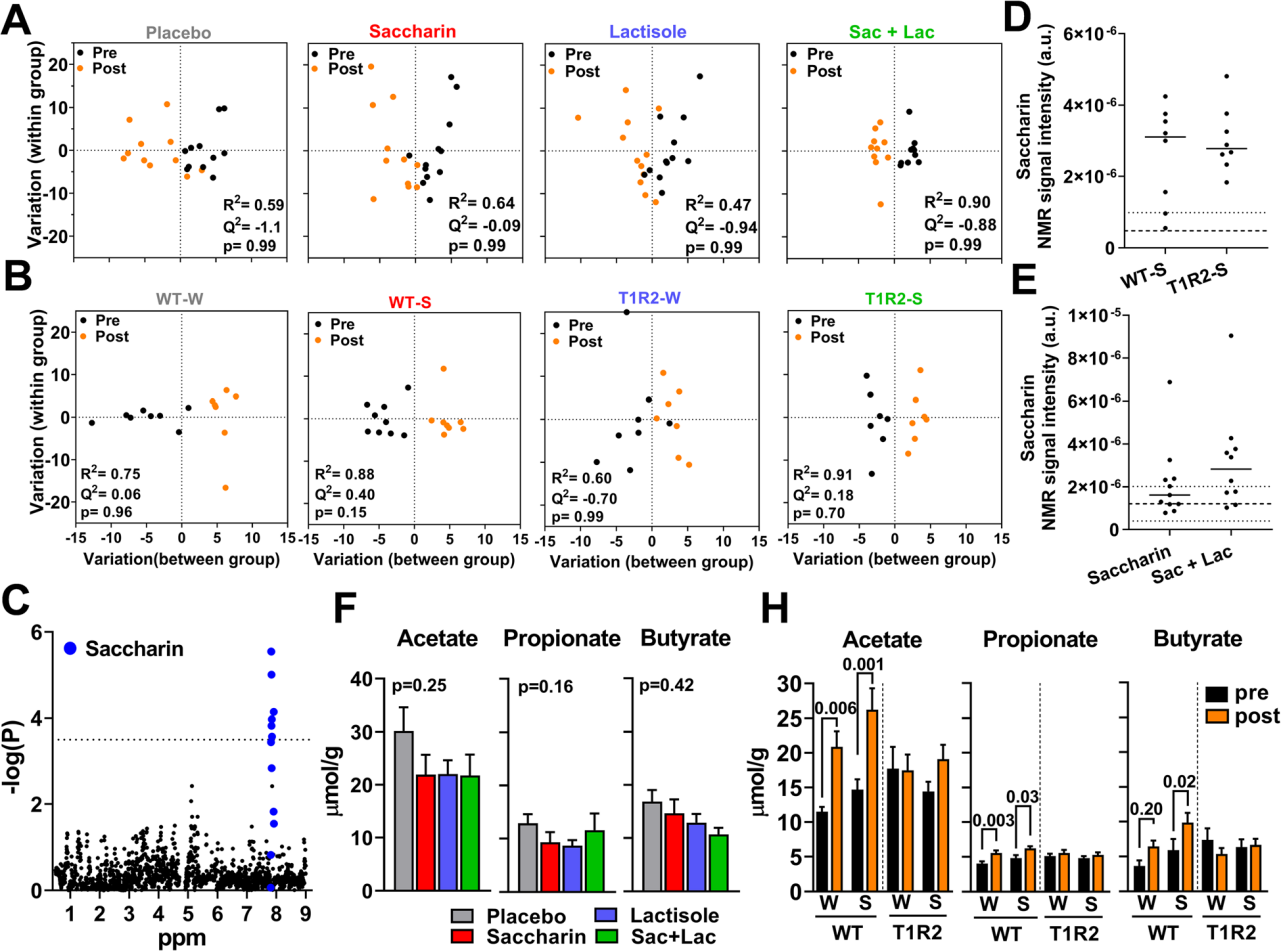
Treatment effects on fecal metabolomics in humans and mice. **a** Pre-post treatment variation in fecal metabolites within each treatment group in humans and **b** in mice using orthogonal partial least squares discriminant analyses (OPLS-DA). **c** Statistical significance (-log(p)) of pre-post treatment differences (Δ) in NMR spectral bins (ppm) of fecal metabolites in mice. In blue, NMR spectral bins assigned to saccharin. Dotted horizontal line shows statistically significance for the FDR-corrected *p* value. **d** Presence of saccharin in mouse and **e** human post-treatment fecal samples. Dashed lines represent average noise ± SD (detection threshold). **f** Assessment of short-chain fatty acids (SCFA) in human fecal samples post treatment. **h** SCFA in mouse fecal samples pre- and post-treatment (*n* = 8/group). For (**a**, **b**), R2; Q2; and CV-ANOVA *p* value. For **f**, one-way ANCOVA *p* value, pre-treatment as covariate. For **h**, two-way ANOVA repeated measures with post hoc *p* value. W water, S saccharin

Although the interventions did not induce substantial shifts in the gut microbial communities in humans or mice, we tested whether saccharin might have instead altered the intestine’s metabolic profile by performing untargeted metabolomics of fecal samples. Multivariate analysis using Orthogonal Projections to Latent Structures Discriminant Analysis (OPLS-DA) modeling [67] showed that human participants had similar NMR-based metabolomics profiles at baseline (Supp. figure 8A) and none of the interventions affected the fecal metabolome (Fig. 5a). In addition, we did not observe significant changes in the abundance of major metabolites in response to any treatment (Supp. table 11).

In mice, fecal metabolome before the treatment was similar between groups (Supp. figure 8B), but when mice were clustered according to genotype, we found a significant effect on the OPLS-DA (Supp. figure 8C) which was further evaluated by analysis of the OPLS-DA loadings S-plot to identify NMR (i.e., spectral bins) features that account for these differences. We identified a handful of significant NMR peaks in T1R2-KO samples which corresponded to the short-chain fatty acids (SCFA), acetate and butyrate (Supp. figure 8D), but once all spectral features assigned to these metabolites were accounted to calculate relative abundances, no statistically significant differences between genotypes were noted (Supp. table 11). Similar to humans, saccharin did not cause changes in fecal metabolite profiles of mice, independent of genotype (Fig. 5b). However, we found significant differences in spectral peaks assigned to saccharin (Fig. 5c) although no other changes in individual fecal metabolites were observed between the water- and saccharin-treated mice (Supp. table 11). Indeed, saccharin was present in the feces of most mice that consumed the high daily dose of pure saccharin in the water for 10 weeks (Fig. 5d). Similar, but less pronounced, trends were also noted in the feces of several human participants who were assigned to consume saccharin (Fig. 5e). These data clearly indicate that the saccharin dose was sufficient to reach the intestinal microbiota in both mice and humans. In addition, we specifically assessed fecal glucose content in human and mouse samples, but found no treatment or genotype differences, excluding major defects in glucose absorption (Supp. figure 8E). Finally, we independently measured SCFA in feces using LC/MS and found no treatment effect in human participants (Fig. 5f). However, we noticed an age-dependent increase in SCFA in WT mice, but interestingly these effects were absent in T1R2-KO mice (Fig. 5h).

## Discussion

The misperception and concern about the general safety of NCAS can be attributed, in part, to the amount and quality of the available evidence. A critical knowledge gap has been the lack of interventional studies designed to rigorously investigate whether consumption of saccharin, and of other NCAS, is sufficient per se to alter gut microbiota and cause deterioration of glucose homeostasis in healthy individuals. Using a randomized, placebo-controlled design, we clearly show that daily consumption of pure saccharin for 2 weeks at maximum ADI is inadequate to alter fecal microbiota and metabolites or affect glucose tolerance in healthy participants. Notably, identical results were recapitulated in chow fed mice that consumed pure saccharin for 10 weeks equal to 4-times the human ADI.

Over the past 30 years, a number of cross-sectional and observational studies have reported positive correlations between NCAS consumption and outcomes such as T2DM and metabolic syndrome. These findings have alarmed both consumers and health care professionals [22], despite the fact that health and other lifestyle-related characteristics of the populations might have influenced these outcomes. To that end, meta-analyses of epidemiological studies have directly addressed these issues [68]. A systematic review of 10 cohort studies has found that NCAS consumption increased the risk of T2DM by 25%, but this was reduced to 8% when adjusting for obesity [69]. A later meta-analysis of 9 prospective studies showed that the highest quantile of NCAS intake had a 14% increase in the incidence of T2DM, but this association diminished after imputation of missing studies, suggesting a possible publication bias towards positive associations [70]. Findings from these meta-analyses do not provide proof that the consumption of NCAS is innocuous to metabolic regulation, but point out the need of well-designed interventional studies that address specific hypotheses after careful control of extraneous variables. Unfortunately, a paucity of well-controlled interventional studies has further convoluted the subject.

Besides our report, only 3 other interventional studies in humans have assessed the effects of saccharin supplementation on glycemic control [24, 35, 37]. The elegant report by Suez et al [37], which mainly used mice, was first to establish a causative relationship between the consumption of saccharin and the development of glucose intolerance through direct modification of gut microbiota composition. However, in their human cohort, which lacked a control group, only 4 out of 7 treated participants developed glucose intolerance in response to 6 days of saccharin feeding [37]. In contrast, we blindly exposed a total of 23 healthy lean participants (in 2 separate cohorts: Saccharin, or Saccharin plus Lactisole groups) to 15 days of daily consumption of pure saccharin at maximum ADI levels. The treated subjects, who were not regular NCAS users, did not develop glucose intolerance as a group or individually and showed no altered endocrine responses during an OGTT. It is plausible that the effects of saccharin supplementation may be lagging, but the OGTT responses remained unaltered after 2 additional weeks of placebo treatment following the main intervention. In agreement with our findings, 12 weeks of saccharin supplementation using sweetened beverages did not change glucose tolerance (OGTT) in healthy overweight or obese individuals, despite an increase in their body weight [35]. Also 6 weeks of daily saccharin supplementation in patients with T2DM did not change fasting glucose or insulin, but an OGTT was not assessed [24]. Notably, there are no published interventional studies using diabetic participants that have reported a negative effect of NCAS consumption (aspartame, sucralose, or steviol glycosides) in fasting glycemia [25, 27, 29, 31] or in OGTT responses [23]. This may indicate that NCAS intake cannot further deteriorate glucose homeostasis in this population, or it may also suggest that the development of glucose dysregulation in response to NCAS intake may require additional risk factors.

For instance, inter-individual differences in gut microbiota have been shown to affect host responsiveness to dietary interventions [71]. In this regard, the saccharin responders in Suez et al. [37] were retrospectively found to have significantly different baseline microbiome as a group, compared to non-responders. Particularly, prior to the intervention, the relative abundance of RF32 and YS2 taxa (order) was almost absent in the non-responder group (< 1%), but significantly elevated (3–4% of total taxa) in the responder group. In agreement with the prevalence in non-responders, the average relative abundance of these taxa in our cohorts was < 0.5% and this was not altered by saccharin or any other treatment (see Supp. table 5). Notably, these taxa are associated with dietary and disease conditions in rodents and humans. RF32 is elevated in rats fed HFD [72] and, together with YS2, correlate with HFD-induced reduction of intestinal crypt depth in mice [73]. RF32 is also associated with a mouse model of Crohn’s disease [74] and with non-alcoholic fatty liver disease in humans [75]. Together, RF32 and YS2 are also biomarkers of a mouse model of multiple sclerosis [76]. However, whether the higher abundance of these taxa in the responder group at Suez et al. [37] directly influenced the outcome after saccharin treatment is only speculative at this point, since their direct involvement was not studied. Our randomized participants had comparable gut microbiota diversity and composition prior to the interventions. This was partially accomplished by careful consideration of participation criteria which, among other things, required dietary habits within the typical macronutrient intake of the US adults [77, 78].

Regardless, saccharin treatment in our studied population neither altered gut microbiota diversity and composition compared to other interventions, nor it induced any relative changes in the treated participants (i.e., within-subject pre-post analysis). In contrast, Suez et al. [37] reported that the saccharin-induced dysbiosis was substantiated by changes in the abundances of *Bacteroides fragilis*, *Weissella cibaria*, and “*Candidatus* Arthromitus” (species), but the abundance of these taxa was not affected by saccharin in our studies. In agreement with the null effects of saccharin, consumption of formulated sucralose for a week also did not affect gut microbial profiles or glucose tolerance (OGTT) in healthy adults [34]. In this randomized placebo-controlled study, the microbiome composition remained stable in response to placebo or sucralose intervention (within-subject comparisons), but it was noted that, prior to the treatment, the average relative abundance of Firmicutes (phylum) was higher at the placebo vs. the sucralose group, by chance [34]. Thus, applying the same reasoning as above, it is possible that these unexpected differences in microbiota profiles before the treatment might have influenced the post-treatment outcomes of sucralose. To our knowledge, no other interventional study in humans have explored the relationship between the consumption of any other NCAS and gut microbiota, but a cross-sectional study conducted between consumers and non-consumers of aspartame and/or aceK also showed no differences in microbiota composition and function between groups [79]. Therefore, it is still unclear from the small number of interventional studies in humans whether short-term consumption of saccharin and other NCAS can independently induce dysbiosis or any changes in gut microbiota.

Beyond Suez et al. [37], three other interventional studies have reported potential negative effects of NCAS consumption on glucose control in apparently healthy participants [38–40]. Sucralose consumption for 4 weeks reduced acute insulin response (AIR) and the Matsuda insulin sensitivity index derived from an intravenous GTT (IVGTT) and an OGTT, respectively. Surprisingly, blood glucose responses in the IVGTT or the OGTT were not different among the treatment groups [38]. Similarly, daily consumption of 3 sucralose (Splenda) sachets (which also contains dextrose and maltodextrins) for 2 weeks reduced insulin sensitivity index (Si) derived from a modified frequently sampled IVGTT (FSIVGTT), but none of the other surrogate indices were affected (AIR, disposition index, or glucose effectiveness) [39]. Finally, Dallenberg et al. [40] reported that, after 2 weeks of treatment, the combination of sucralose and maltodextrin, but not sucralose alone, reduced insulin secretion (Δ from baseline) compared to sucrose treatment, but similar to the two previous reports [38, 40], OGTT responses were unaffected. Although these studies [38–40] raise the possibility that sucralose intake may moderately alter insulin secretion in healthy adults through synergistic effects with other co-ingested sugars, the absence of accompanied effects on glucose tolerance (OGTT or IVGTT) severely limits their clinical significance [80]. This conclusion is further supported by a large number of interventional studies which, similar to ours, found that glucose tolerance (OGTT) is not affected by the consumption of sucralose [27, 32, 34–36] or any other NCAS studied [23, 33, 35, 36].

The absence of effects following short-term saccharin supplementation in our human study cannot exclude the possibility that the deleterious consequences of saccharin consumption might require higher doses and/or longer duration. Because of safety limitations regarding the dose and duration of treatment involving human participants, we supplemented C57Bl\6J mice with pure saccharin for 10 weeks using a target dose that exceeded the human ADI by 4 times adjusted for mouse body surface area to discern possible mechanistic effects that might have not been apparent in the human study. Surprisingly, but in agreement with the human findings, glucose tolerance, gut microbiota diversity, and composition were unaffected by the higher saccharin dose and extended duration of the treatment in chow fed mice. Also, no differences in intestinal glucose transport, gut permeability, or glucose malabsorption were noted, excluding any tampering of chronic saccharin feeding with host-dependent gut functions that could affect glucose excursions. Similar to our findings, a thorough study in C57Bl\6J mice demonstrated that, among other NCAS, saccharin administration alone for 4 weeks did not affect body weight, OGTT responses, or insulin sensitivity (insulin tolerance test) [81]. In contrast, after 12 weeks of saccharin supplementation, chow-fed ICR/HaJ mice showed a marginal increase (15%) in the AUC of glucose during an OGTT, which may be linked to the concomitant increase in food intake and weight gain in the same mice [82]. Our saccharin-fed mice consumed similar amount of chow and experienced the same age-related increases in body weight compared to water control. Nevertheless, Suez et al. [37] convincingly demonstrated, using antibiotic treatments and fecal transplantations, a causative relationship between saccharin-induced glucose intolerance and changes in gut microbiota. Saccharin supplementation for 11 weeks produced extensive changes in intestinal microbiome. Out of more than 40 altered OTUs, the authors highlighted *Clostridiales* (order), *Bacteroides* (genus), and *Lactobacillus reuteri*, *Bacteroides vulgatus,* and *Akkermansia muciniphila* (species) as the main signatures for the saccharin-induced dysbiosis [37]. None of these taxonomic features were altered in our comparisons between saccharin- and water-treated cohorts, or within mice of the saccharin-treated group (pre-post analysis). This is very surprising, but further considerations may shed light on these discrepancies. In this latter study [37], saccharin was provided either as a 10% solution of commercial saccharin in chow-fed mice or as pure saccharin in HFD-fed mice [37]. Commercial saccharin contains 95% glucose by mass. This suggests that based on the reported ad libitum values of water and food intake, these mice received the majority (about 70%) of total daily calories in the form of liquid glucose [37]. Since chow-fed mice on pure saccharin were not included in the design, these accompanied dietary features cannot be ignored considering that glucose consumption or HFD feeding can independently alter the gut microbiome [83]. Instead, we supplemented chow-fed mice with pure saccharin to exclude potential synergistic effects with high dietary glucose or fat and test the independent effects of saccharin feeding on gut microbiota and glucose tolerance.

Although there are no other reports that assess interactions between saccharin consumption, gut microbiota, and glucose homeostasis, a few mouse studies have explored the effects of saccharin intake on gut microbiome alone. Saccharin treatment for 6 months was associated with changes in *Ruminococcus, Adlercreutzia, Dorea, Corynebacterium, Roseburia,* and *Turicibacter* (species) [48] and the detection of liver inflammatory markers, so it unclear whether this microbial signature is a de facto saccharin-induced effect [84]. To further add to the complexity of the subject, saccharin administration ameliorated the outcomes of dextran sodium sulfate (DSS)-induced colitis through beneficial bacteriostatic effects [85]. Interestingly, our saccharin-treated mice had a moderate decrease in the Bray-Curtis dissimilarity (pre- vs. post-treatment) compared to water-treated mice. Since no other taxonomic changes were observed, this finding suggests that in chow-fed mice, saccharin treatment induced less overall changes in microbial composition over time. This is consistent with the bacteriostatic effects of saccharin [85, 86], but in contrast to the noticeable changes described by others [37, 48]. Generally, the lack of a common saccharin-induced microbiota signature and the variable physiological outcomes among these reports may reflect differences in the diet or the health status of the treated mice. This further indicates that the type and magnitude of effects following saccharin consumption may be intimately linked to these factors.

Evidence from studies that use other NCAS (sucralose, aceK, or neotame) also point in this direction. To our knowledge, all published studies exploring interactions of NCAS consumption and gut microbiota have reported an effect [49–52, 87, 88]. However, it is noticeable that, among these numerous studies, there is no reproducible microbiota signature even for commonly studied NCAS, such as sucralose [49, 51, 52, 87]. The consequences of these microbiota alterations on glucose tolerance were not assessed in these reports, except for one that found no effect on glucose tolerance (OGTT) after 6 weeks of sucralose supplementation in ileitis-prone SAMP mice [52] that is consistent with other independent reports which showed that consumption of NCAS, such as sucralose or aceK, has either a minor [82], or no effect [81] on glucose tolerance.

Although gut microbiota abundances were unaltered by the treatments in our studies, changes in microbial function and metabolism [89] might predispose the host to dysbiosis [90]. Our findings do not support this possibility either, since saccharin, or any other treatment, did not alter fecal metabolite profiles in humans and mice. The microbiota-induced pathophysiology is often linked to changes in microbial production of SCFA [91], but saccharin did not alter fecal concentrations of SCFA, mirroring the null effect observed in untargeted metabolite profiles. However, the age-dependent increase in SCFA in the WT mice is consistent with the age-dependent development of glucose intolerance in the same mice and it is in agreement with findings showing that increased fecal SCFA correlate with age, obesity, and metabolic dysregulation [92]. Notably, in T1R2-KO mice, the absence of SCFA increases with aging correlates with the absence of glucose intolerance. These associations require further investigation since fecal concentrations of SCFA can be affected by several factors including transit time [93] and colonic clearance [94].

Interestingly, saccharin was detected in the feces of several saccharin- or saccharin plus lactisole-treated participants and in the feces of almost all treated mice, confirming saccharin’s bioavailability for microbial metabolism. From a clinical perspective, this observation is very significant because about 90% of ingested saccharin is absorbed in the small intestine and eliminated in the urine without biotransformation, while the remainder is excreted in the feces [95]. Thus, only a very small portion of ingested saccharin can reach and potentially be metabolized by the microbes at the large intestine. In humans, we administered saccharin equivalent to the ADI [56], suggesting that saccharin bioavailability was not a limiting factor for gut microbes in our population. Nevertheless, even in high saccharin consumers (> 90th percentile), the average intake is only about 2 mg/kg/d, a minor fraction of the ADI (5 mg/kg/d) [96]. Taken together, it is therefore unclear how typical saccharin use can practically alter the gut microbiota of a healthy consumer.

NCAS are bona fide ligands for STRs, so it is reasonable to speculate that if consumption of saccharin and of other NCAS alter glucose homeostasis, a common underlying mechanism involving the host might exist. Thus, a secondary aim of our studies was to test whether STR partially mediate the effects of NCAS feeding. Participants that consumed lactisole, a human-specific inhibitor of STRs, or mice with genetic ablation of STRs had no differences in glucose tolerance or gut microbiota in response to saccharin feeding. This suggests that, in the absence of a primary effect of saccharin consumption, the role of STR signaling is either obscured, irrelevant, or untestable. Nevertheless, we observed a genotype effect in mice independent of treatment. T1R2-KO mice had reduced IG.GTT responses and ex vivo glucose transport compared to WT littermates, confirming our previous findings [58]. Interestingly, although WT mice developed mild age-related glucose intolerance, T1R2-KO mice were resistant to these effects. We previously showed that T1R2-KO mice were also protected against metabolic derangements induced by HFD [97], suggesting that STR signaling may be involved in age- and diet-dependent deterioration of glucose homeostasis.

Although we report no adverse effects of short-term saccharin consumption on glucose tolerance in healthy lean participants and mice, our study has some notable limitations. First, we tested saccharin as a representative NCAS, but it is unknown whether our results can be extrapolated to all NCAS. Since the six FDA-approved NCAS have different metabolic fates and bioavailability [98], potential health implications relevant to their consumption must be addressed separately. Second, the duration of treatment in humans was limited to 2 weeks, which may have been inadequate to induce physiological effects in a healthy young population. This does not preclude the possibility that years of chronic saccharin use may eventually lead to slow maladaptive responses or predispose consumers to the development of disease. Third, we focused on a number of outcomes based on previous reports and specific objectives. Thus, saccharin might have altered other physiological parameters that, if measured, may have helped identify other adverse health conditions linked to NCAS consumption. Finally, we acknowledge that the relatively small sample size in our study may have limited statistical sensitivity. Our study was powered (> 80%) to detect differences in glucose tolerance based on previous findings [37] and that metric was met. Also, in addition to between group comparisons, we performed within group analysis (pre-post) to circumvent inter-individual variability in microbiome and even performed pairwise comparisons between the placebo and saccharin group only. Regardless of the statistical approach, the observed size effect of saccharin treatment was small and indistinguishable from the placebo treatment. Nevertheless, to unveil possible effects of chronic saccharin consumption, future studies should be specifically designed to evaluate subtle size effects over longer treatment periods.

## Conclusions

We clearly show that short-term saccharin supplementation per se is insufficient to alter gut microbiota or induce glucose intolerance in apparently healthy humans and mice consuming typical ad libitum diets. The clinical significance of our null findings should not be underestimated since it emphasizes that the recommended saccharin use is likely safe for healthy consumers that wish to substitute dietary sugars for weight management or caloric control. Most importantly, our null findings do not necessarily contradict previous reports showing some harmful metabolic effects of NCAS intake. Together, they highlight that high NCAS consumption may exert negative health outcomes accommodated by other physiological or dietary parameters [99]. Therefore, for many individuals, such as those studied here, consumption of NCAS is likely innocuous. For some others, however, it may be harmful and thus justify revisions in health policy that guides optimal NCAS use [100]. Consequently, it is imperative that future interventional studies concentrate in isolating and identifying the underlying physiological or lifestyle conditions that potentially makes NCAS use harmful.

## Methods

### Experimental design

#### Human studies

We conducted a randomized, placebo-controlled, double-blind, interventional study (NCT02835859) at the Advent-Health Translational Research Institute (TRI) in healthy lean male and female participants who were randomly assigned to four intervention groups. Recruitment, enrollment, and all study-related visits, including specimen collection and point-of-care laboratory testing, took place at Advent-Health. Subjects were recruited between January 2017 and February 2018. The study was approved by the Institutional Review Board at Advent-Health and all participants signed an informed consent.

Healthy men and women 18–45 years of age were recruited from volunteer lists and by social media to participate in the study. Only subjects who consumed less than a can of diet beverage or a spoonful of NCASs weekly (or the equivalent from foods) during the past month, whose body mass index (BMI) ≤ 25.0 kg/m^2^, and who were weight stable (± 3 kg) during the 3 months prior to enrollment were included. Subjects with acute or chronic medical conditions that would contraindicate participation in the research testing or that were taking medications that could potentially affect metabolic function were excluded. Specifically, individuals with diabetes, bariatric surgery, inflammatory bowel disease, or a history of malabsorption and pregnant or nursing women were excluded. A complete list of inclusion and exclusion criteria is available (Supp. methods).

Participants were randomized into four treatment groups and were instructed to consume capsules containing (1) pulp filler/placebo (1000 mg/day 1) sodium saccharin (400 mg/day), (3) lactisole (670 mg/day), or (4) sodium saccharin (400 mg/day) + lactisole (670 mg/day) twice daily for 2 weeks. A sealed envelope with the randomization allocation sequence (SAS procedure PROC PLAN) was given to the pharmacist who prepared and provided the appropriate treatment. The pharmacist was the only un-blinded member of the study. Diet-related instructions were provided to avoid additional consumption of NCASs for the duration of the study. Participants were asked to give blood samples and stool samples during their visits. The investigation agents, saccharin and lactisole, were formulated in capsules for oral delivery (Compounding Pharmacy, Advent-Health) at the maximum acceptable daily intake (ADI) [56]. Capsules were made of hard gelatin (appropriate for dry ingredients in powder form) following USP 795 requirements. Once ingested they quickly disintegrate in the stomach releasing their content. A schematic of the experimental design is shown in Supp. Figure 5A. At visit 1 (pre-intervention), participants arrived at the TRI after a 10-h overnight fast omitting breakfast and the following procedures were performed: (1) stool sample collection, (2) assessment of dietary compliance, (3) vital signs, (4) measurements of weight, (5) insertion of an intravenous (IV) catheter for blood draws, (6) baseline blood sampling (*t* = − 10, 0 min), (7) oral consumption of a 75 g glucose solution (300 mL) to assess glucose tolerance (i.e., OGTT), (8) OGTT blood sampling (*t* = 10, 20, 30, 45, 60, 90, 120, 180 min), and (9) participants were provided with a 2-week supply of treatment capsules and were instructed to consume 2 capsules a day (morning and evening) with water until the night before their next visit. At visit 2 (post-treatment), the same procedures as listed above were repeated. All groups were subjected to additional 2 weeks of pulp filler/placebo capsule treatment (blinded for participants), and at visit 3 (recovery), the same procedures were performed.

The blood was collected in K_2_EDTA tubes with a cocktail of protease, esterase, and DPP-IV inhibitors (BD^TM^ P800 blood collection system; BD Bioscience, CA). Glucose concentrations were measured by a point of care device (NOVA StatStrip Meter); insulin, C-peptide, total GLP1, and glucagon concentrations by immunoassay (Milliplex Map Kit, Millipore, MA).

#### Mouse studies

All animal experimental procedures were approved by the Institutional Animal Care and Use Committee (IACUC) committee of The Ohio State University. Whole body T1R2-deficient mice (T1R2-KO; a gift of Dr. Zuker) were used with WT littermates back-crossed on the C57Bl\6J strain for at least 10 generations. After weaning, all mice were housed individually in ventilated caging with limited shared environmental exposure and placed on standard polysaccharide chow diet (Teklad #2016) for 4–5 weeks. Eight week-old mice were randomly assigned to one of the following treatment groups for additional 10 weeks (Supp. Figure 5B): (1) drinking water only (control) and (2) drinking water plus saccharin. All groups were on standard chow diet, and saccharin concentrations were adjusted based on pilot studies aiming to (a) avoid taste aversive effects (< 0.3% saccharin in water) [101], (b) ensure equal consumption between genotypes since WT mice can taste saccharin but T1R2-KO cannot, and (c) to achieve an average daily dose equal to 4 times (250 mg/kg), the human ADI (62 mg/kg) adjusted for mouse body surface area [57]. An intra-gastric GTT (IGGTT) was performed at baseline, week 2 and week 10 of the intervention. Fecal pellets, we collected at baseline and at week 10 of the intervention for each mouse. The IGGTT was performed in 5-h fasted mice (h) which received 1 g/kg body weight (BW) of glucose. For the saccharin-treated groups, saccharin was maintained in the drinking water during the fasting period prior to testing. A baseline IGGTT was performed the day following the initiation of the interventions to account for possible acute effects of saccharin feeding on the test. Blood glucose was sampled from the tail and analyzed with an AlphaTRAK blood glucose monitoring meter (North Chicago, IL). Glucose tolerance curves over time are shown in absolute values. Area under curve (AUC) was calculated using the trapezoid method adjusted for fasted baselines.

### Ussing chamber

Ex vivo glucose transport was measured in intact intestinal sections by monitoring short-circuit current and measuring ^14^C isotopic flux of 3-O-methyl-glucose ([^14^C]-3-OMG), exactly as described previously [58]. To assess gut permeability, 0.2 mg/ml of 4 kDa FITC-dextran (Sigma) was added to the donor chamber of pre-equilibrated jejunums and FITC flux to the acceptor side was assessed every 15 min for 1.5 h in a fluorimeter at 485 nm excitation and 528 nm emission.

### Fecal microbiota

Genomic DNA was isolated from mouse and human feces using QiaAmp DNA stool kit (QIAGEN), with an additional step of bead beating for 5 min with 0.1 mm beads to ensure maximum lysis of bacterial cells. Multiplexed libraries were prepared according to the protocol from Illumina using V3–V4 regions of 16S rRNA and HiFi HotStart DNA Polymerase (Kapa Biosystems) for amplification. Final amplified products were quantified by ABI Prism library quantitation kit (Kapa Biosystems). Each sample was diluted to 10 nM, and equal volume from each sample was pooled. The quality of the library was checked by Bio-Rad Experion bioanalyzer (Bio-Rad). Illumina MiSeq platform was used for sequencing (Novogene Bioinformatics Technology Co., Ltd).

Raw FASTQ sequences were quality checked with FastQC v0.11.5. Raw sequences were trimmed with “cutadapt” v2.6 to remove low-quality bases and adaptor sequences. The trimmed FASTQ files were converted into a Qiime2 v2019.1 file format. The imported forward and reverse reads were merged using “vsearch” with a minimum sequence length of 200 base pairs. Joined pairs were quality trimmed using Qiime2 “quality filter” with an average quality score of 20 (Q20) over a 3 base pair sliding window and removing trimmed reads having less than 75% of their original length. “Deblur 16S rRNA positive filter” was used as a final quality control step by dereplicating and removing chimera sequences from each sample; reads were trimmed to a final length of 195 base pairs. Taxonomic analysis and Operational Taxonomic Unit (OTU) tables were created with Qiime2 using 100% sequence identity threshold [102] and converted using biom format. The median sequencing depth for human samples was 51,274 reads and for mouse samples 48,561 reads. To avoid bias of sequencing depth, the OTUs were filtered for singletons and rarefied to lowest sample depth (39,895 for humans and 5863 for mouse) resulting in a Good’s coverage index > 99.98% for all human or mouse samples (MicrobiomeAnalyst.ca [103]). Next, alpha diversity was calculated using the following indices: Chao1 (species richness), and Shannon and Simpson (species richness and evenness) [63, 64] (MicrobiomeAnalyst.ca). Paired pre-post (within subject) and between treatment analysis was assessed using repeated measurements ANOVA after performing normality tests (Graphpad Prism v8). Next, OTUs with very small counts (< 4) in very few samples (< 20% prevalence) were filtered out from all subsequent analysis [103]. For beta diversity analysis, Bray-Curtis dissimilarity was calculated using the R package Vegan [104] and UniFrac distances were calculated with the R package GUniFrac [105]. The dissimilarity matrix was ordinated using Nonmetric Multidimensional Scaling (NMDS), and the between-subject beta diversity was tested for statistical significance with permutational Multivariate ANOVA (PERMANOVA, Adonis) [106]. Within-subject Bray-Curtis dissimilarity and UniFrac distance comparisons between treatments were assessed using ANOVA or Kruskal-Wallis tests after performing normality tests. Linear Discriminative Analysis (LDA) Effect Size (LefSE) was used to unveil discriminative features between and within treatments using the Galaxy workflow framework (https://huttenhower.sph.harvard.edu/galaxy/) [65]. Bacterial community compositions at each taxonomic rank (phylum, class, order, family, genus, and species) were retrieved (MicrobiomeAnalyst.ca) and scaled into the Euclidean space with Centered Log-Ratio (CLR) transformation [107] to calculate within-subject pre-post treatment compositional differences (Δ) which were then evaluated by ANOVA (Metaboanalyst.ca). The size effect of the saccharin treatment was calculated using Cohen’s *d* at the genus level [66].

### Fecal metabolomics

The nuclear magnetic resonance (NMR) spectra of aqueous fecal extracts were acquired at 298 K on a Bruker Avance III 800 MHz spectrometer equipped with a TCI probe (Bruker Biospin, Germany). The ^1^D ^1^H NMR experiments were conducted using the first increment of the nuclear Overhauser enhancement spectroscopy (NOESY) pulse sequence with presaturation for water suppression (Relaxation delay-90-t1-90-mixing time-90-Free induction decay). The acquisition parameters were as follows: 64 scans and 4 dummy scans, 64 K data points, 90° pulse angle (11.3 us), relaxation delay of 3 s, and a spectral width of 14 ppm. The spectra were acquired without spinning the NMR tube in order to avoid spinning side band artifacts. The free induction decays were multiplied by a decaying exponential function with a 1 Hz line broadening factor prior to Fourier transformation. The ^1^H NMR spectra were corrected for phase and a polynomial fourth-order function was applied for base-line correction. Chemical shifts are reported in ppm as referenced to Trimethylsilylpropanoic acid (*δ* = 0). NMR signal were assigned using a range of 2D NMR spectra, namely ^1^H − ^1^H correlation spectroscopy, ^1^H − ^1^H total correlation spectroscopy, ^1^H − ^13^C edited heteronuclear single quantum correlation, and ^1^H − ^13^C heteronuclear multiple bond correlation spectra. 1D and 2D NMR spectra were processed using TopSpin 3.2 (Bruker Biospin, Germany). The spectral region δ 0.50–10.0 was integrated into regions with equal width of 0.005 ppm using the AMIX software package (V3.8, Bruker-Biospin). The region δ 4.70–4.90 was discarded due to imperfect water saturation. Prior to statistical data analysis, each bucketed region was normalized to the total sum of the spectral intensities to compensate for the overall concentration differences.

Multivariate statistical analysis was carried out with SIMCA-P+ software (version 14.1, Umetrics, Sweden). Data were mean-centered and scaled using the Pareto method, while log-transformation was applied to achieve an improved normal distribution of the data. Principal component analysis (PCA) and orthogonal projection to latent structures with discriminant analysis (OPLS-DA) were conducted on the scaled data [67]. The OPLS-DA model’s confidence level for membership probability was set to 95% and was validated using a 7-fold cross validation method. The quality of the model was assessed by the values of R^2^Y and *Q*^2^. The R^2^Y metric describes the percentage of variation explained by the model; *Q*^2^ shows the predictive ability of the model and is expected to be > 0.5. The difference between these metrics, which is expected to be < 0.3, describes the model’s fitness. The significance of the OPLS-DA models was further tested with an ANOVA of the cross-validated residuals (CV-ANOVA) [108].

### Fecal short chain fatty acids

Liquid chromatography tandem mass spectrometry (LC-MS/MS) methods for SCFA were performed as described [109]. Briefly, samples of mouse and human feces were thawed on ice. Samples were then homogenized in 50% acetonitrile, containing ^13^C-propionate as an internal standard at a ratio of 10 μL solvent per 1 mg fecal sample. Fecal samples were then derivatized as described previously [109]. Samples were sealed and stored at 4 °C until analyses and throughout LC-MS/MS quantification. All LC-MS/MS analyses were performed within 24 h of sample creation. Samples were analyzed on an Agilent 6460 QQQ LC-MS/MS system, using a Poroshell EC-C18 column (3.0 × 50 mm). Collision energies were 10 for butyric acid, 5 for propionic acid, and 15 for acetic acid. Retention times and mass transitions for each SCFA monitored were butyrate 7.138 min, 222→137; propionate 5.097 min, 208→165, 208→137; ^13^C propionate 5.097 min, 209→165, 209→137; and acetate 2.754 min, 194→137. SCFA levels were quantified using standard curves generated using authentic standards and normalized using ^13^C propionate as an internal standard. Data was analyzed using the Agilent MassHunter Quantitative Analysis software suite.

### Gene expression

Gene expression of scraped mucosa from mouse intestines was performed as described [58] using the following genes: t1r2 (forward: GAACTGCCCACCAACTACAA, reverse: CCATCGTGGACAGACATGAA), t1r3 (forward: CCAGTGAGTCTTGGCTGACA, reverse: TTCAGTGAGGCACAGAATGC), sglt1 (forward: TGGAGTCTACGCAACAGCAAGGAA, reverse: AGCCCACAGAACAGGTCATATGCT), glut2 (forward: CCCTGGGTACTCTTCACCAA, reverse: GCCAAGTAGGATGTGCCAAT).

### Statistical analyses

For human studies, sample size calculation (PROC GLMPOWER, SAS) was based on the minimal detectable difference of glycemic responses (area under curve) during an OGTT performed before and after 7 days of saccharin treatment (Fig. 4b of reference [37]), using an ANCOVA model with baseline as covariate to provide 80% statistical power for one-sided 0.05 significance level test. Differences between groups in glycemic and hormonal responses (i.e., AUC) during the OGTT were tested via ANCOVA with the baseline AUC as the covariate, followed by post hoc multiple comparisons. To investigate the treatment effect at the different visits, we built repeated measures ANCOVA with treatment, time, and treatment × time interaction as the main effects, along with baseline AUC as a covariate, followed by post hoc multiple comparisons. For mouse studies, differences between groups in glycemic responses during the OGTT and ex vivo intestinal transport and gene expression were tested by two-way ANOVA. A *p* value < 0.05 was considered statistically significant. All analyses will be performed with SAS version 9.4 (SAS Institute Inc.).

## Supplementary Information

**Additional file 1: Figure S1.** Participant inclusion flowchart. **Figure S2.** Longitudinal treatment effects glucose and hormonal excursions during an oral glucose challenge in humans. **Figure S3.** Treatment compliance and intestinal gene expression in mice. **Figure S4.** Pre-treatment comparisons of gut microbial diversity and composition (genus) in humans. **Figure S5.** Pre-treatment comparisons and treatment effects on gut microbial diversity and composition in humans. **Figure S6.** Pre-treatment comparisons of gut microbial diversity and composition (genus) in mice. **Figure S7.** Pre-treatment comparisons and treatment effects on gut microbial diversity and composition in mice. **Figure S8.** Pre-treatment fecal metabolomics in humans and mice. **Figure S9.** Experimental design of the human and mouse studies.

**Additional file 2: Table S1.** Baseline characteristics of participants. **Table S2.** Study compliance. **Table S3.** Detailed statistical analysis of Alpha Diversity Indices of genus and family ranks. **Table S4.** Detailed statistical analysis of Beta Diversity using NMDS plots and Bray Curtis dissimilarity index of the genus and family ranks. **Table S5.** Mean relative abundances of microbial communities and their change in response to treatment. **Table S6.** Linear discriminatory analysis (LDA) effect size (LEfSe) for each treatment (pre-post). **Table S7.** Detailed statistical analysis of Alpha Diversity Indices of genus and family ranks. **Table S8.** Detailed statistical analysis of Beta Diversity using NMDS plots and Bray Curtis dissimilarity index of the genus and family ranks. **Table S9.** Mean relative abundances of microbial communities and their change in response to treatment. **Table S10.** Linear discriminatory analysis (LDA) effect size (LEfSe) for each treatment (pre-post). **Table S11.** NMR-based metabolite changes (post-pre) comparisons between treatment groups. **Table S12.** Pairwise comparison between control (placebo or water) and saccharin treatment in humans and mice.

## Data Availability

The raw sequence data from 16S rRNA gene amplicon sequencing were submitted to NCBI BioProject under accession number PRJNA605207 https://www.ncbi.nlm.nih.gov/bioproject/PRJNA605207

## Abbreviations

3-OMG: 3-O-methyl-glucose
ADI: Acceptable daily intake
ANCOVA: Analysis of covariance
ANOVA: Analysis of variance
AUC: Area under the curve
BW: Body weight
FDA: Food and Drug Administration
GLP-1: Glucagon-like peptide 1
IGGT: Intragastric glucose tolerance test
KO: Knockout
NCAS: Non-caloric artificial sweeteners
NMDS: Non-metric multidimensional scaling
OGTT: Oral glucose tolerance test
OPLS-DA: Ortigonal partial least squares discriminant analysis
OTU: Operational taxonomic unit
SCFA: Short-term fatty acids
STRs: Sweet taste receptors
TRI: Translational research institute (Advent-Health)
WT: Wild-type
