# Supplementary material for: High-dose saccharin supplementation does not induce gut microbiota changes or glucose intolerance in healthy humans and mice
Source: Microbiome. 2021 Jan 12;9:11. doi: 10.1186/s40168-020-00976-w (PMC7802287; doi:10.1186/s40168-020-00976-w)
Supplement: Supplementary file 2 — Additional file 1: Figure S1. Participant inclusion flowchart. Figure S2. Longitudinal treatment effects glucose and hormonal excursions during an oral glucose challenge in humans. Figure S3. Treatment compliance and intestinal gene expression in mice. Figure S4. Pre-treatment comparisons of gut microbial diversity and composition (genus) in humans. Figure S5. Pre-treatment comparisons and treatment effects on gut microbial diversity and composition in humans. Figure S6. Pre-treatment comparisons of gut microbial diversity and composition (genus) in mice. Figure S7. Pre-treatment comparisons and treatment effects on gut microbial diversity and composition in mice. Figure S8. Pre-treatment fecal metabolomics in humans and mice. Figure S9. Experimental design of the human and mouse studies. [file 40168_2020_976_MOESM2_ESM.pdf]

Supp. Fig.1

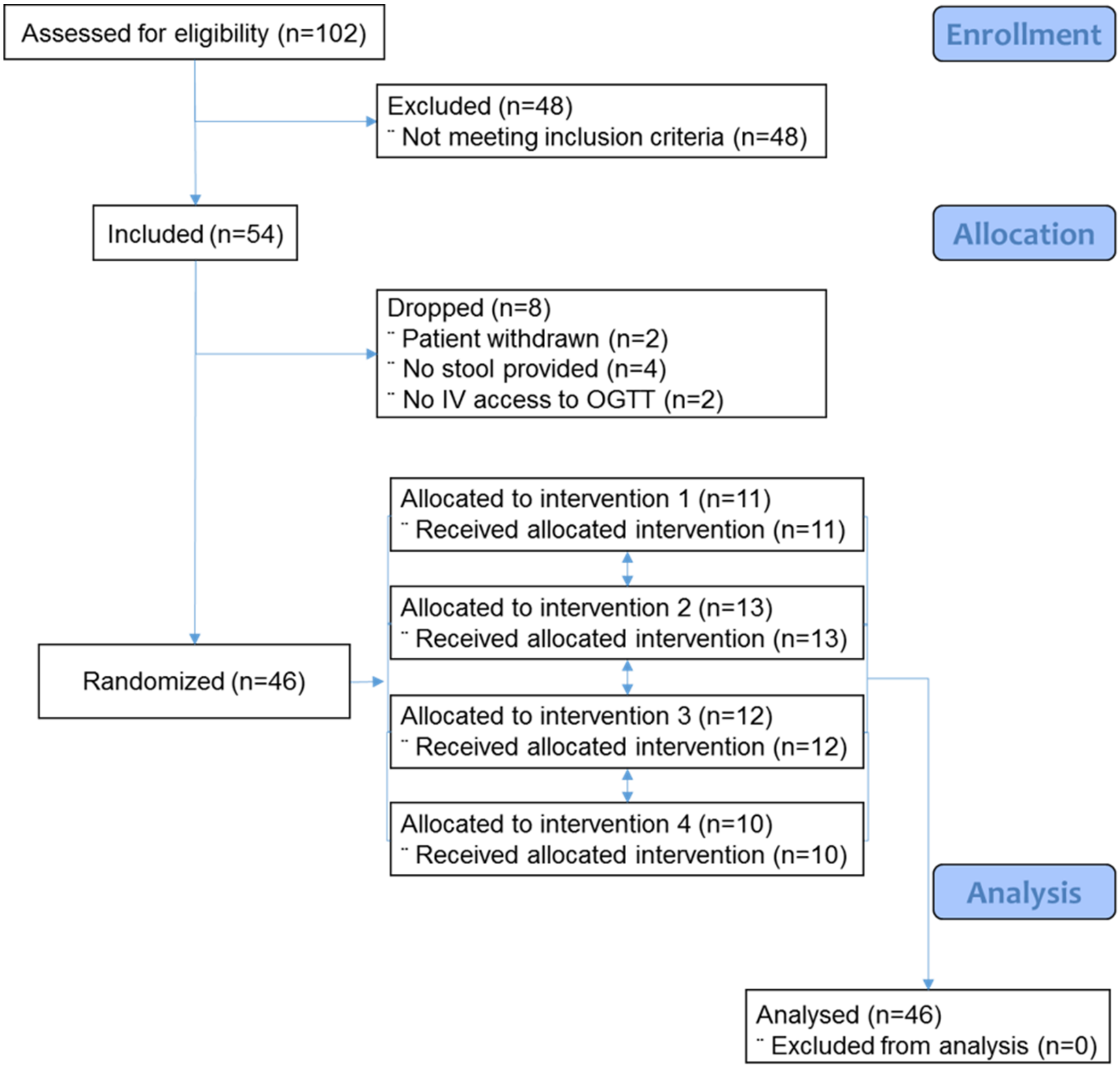

Supplemental figure.1 Participant inclusion flowchart

Out of the 102 screened subjects, a total of 54 were allocated among the four experimental groups. Eight non-compliant individuals were removed from the study at various stages. Forty-six randomized subjects completed the interventions. (Intervention 1: Placebo; Intervention 2: Saccharin; Intervention 3: Lactisole; Intervention 4: Saccharin and Lactisole; IV, Intravenous; OGTT, oral glucose tolerance test).

Supp. Fig.2

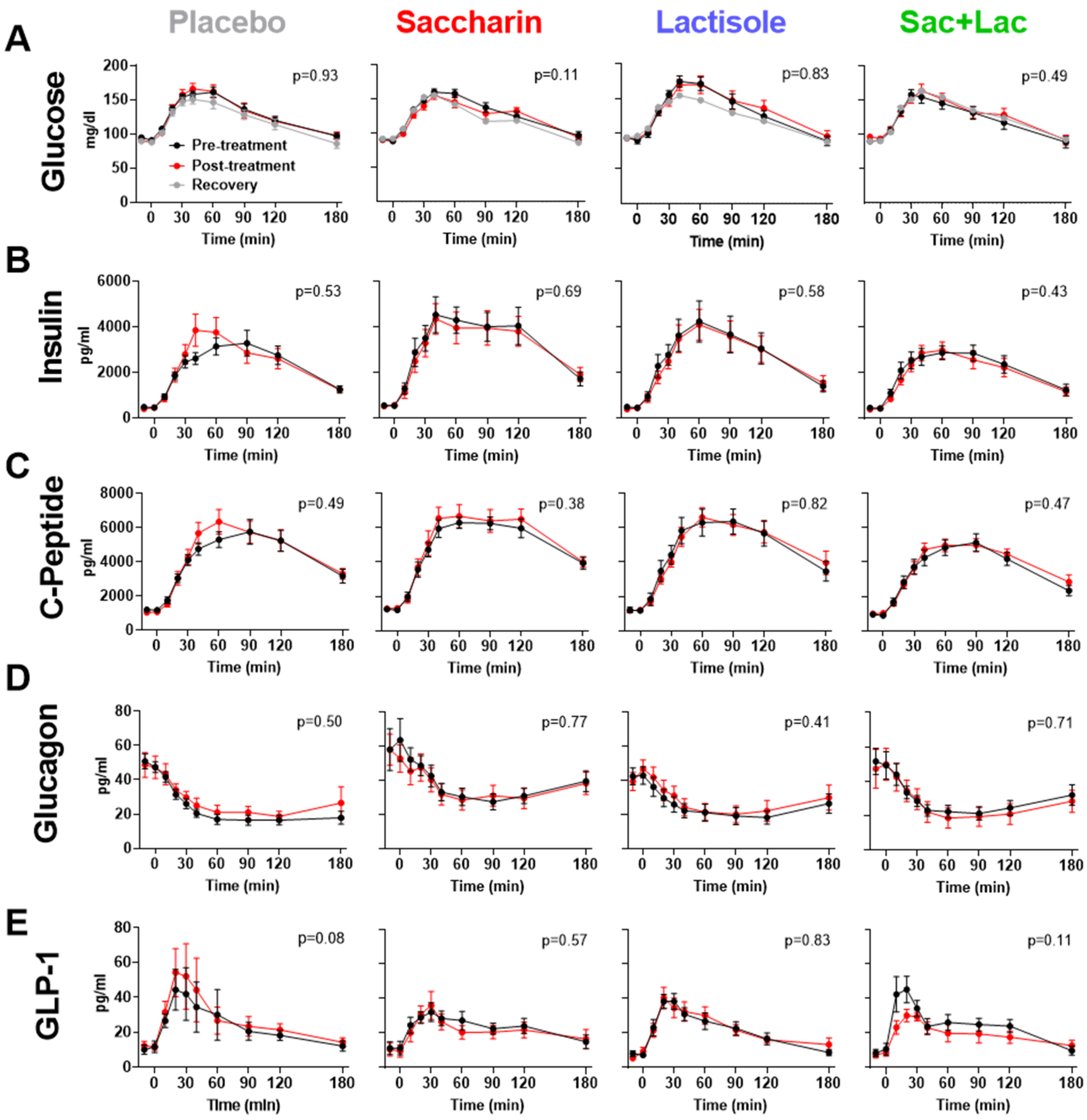

Supplemental figure.2 Longitudinal treatment effects glucose and hormonal excursions during an oral glucose challenge in humans

Plasma excursions of **(A)** glucose, **(B)** insulin, **(C)** C-peptide, **(D)** glucagon, and **(E)** GLP-1 in response to an oral glucose challenge before (pre-treatment) or 2 weeks after (post-treatment) the interventions. For glucose only, 2 weeks wash-out period (recovery) after the main intervention. (n=10-13/group). Two-way ANOVA repeated measures; $p>0.05$ for all comparisons A-E.

Supp. Fig.3

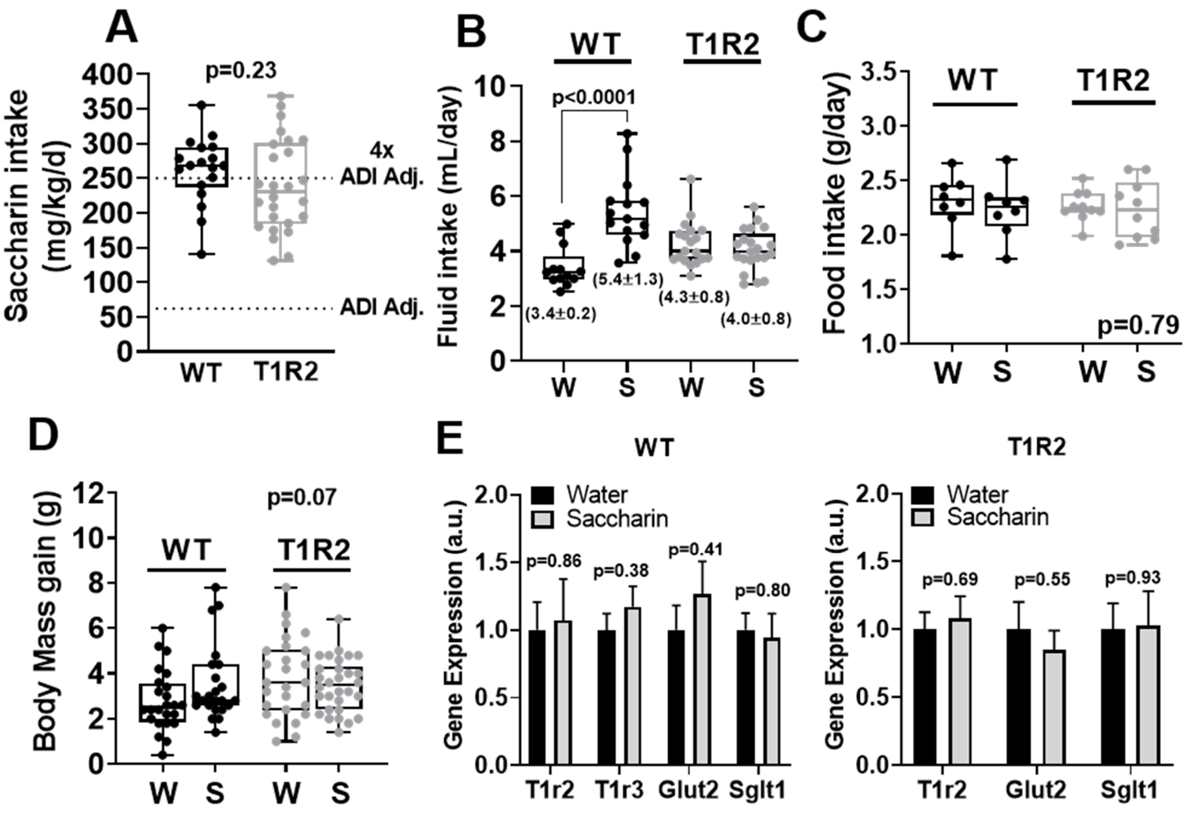

Supplemental figure.3 Treatment compliance and intestinal gene expression in mice

Average daily consumption of **(A)** saccharin, **(B)** fluids and **(C)** food in WT and T1R2-KO (T1R2) mice subjected to 10 weeks of water (W) or saccharin (S) supplementation. In (A), dotted horizontal lines show saccharin consumption equivalent to human ADI and 4x ADI adjusted for body surface. In (B), the value of average (± SEM) fluid consumption per group is shown in parenthesis. **(D)** Body mass gain in response to treatment. **(E)** Gene expression of T1r2, T1r3, Sglt1 and Glut2 in jejunal mucosa of mice following treatment (n=8/group). For, (A and E), Student's t-test. For (B, C and D), Two-way ANOVA p value of genotype x treatment effect; p-value of post-hoc test (B only).

Supp. Fig.4

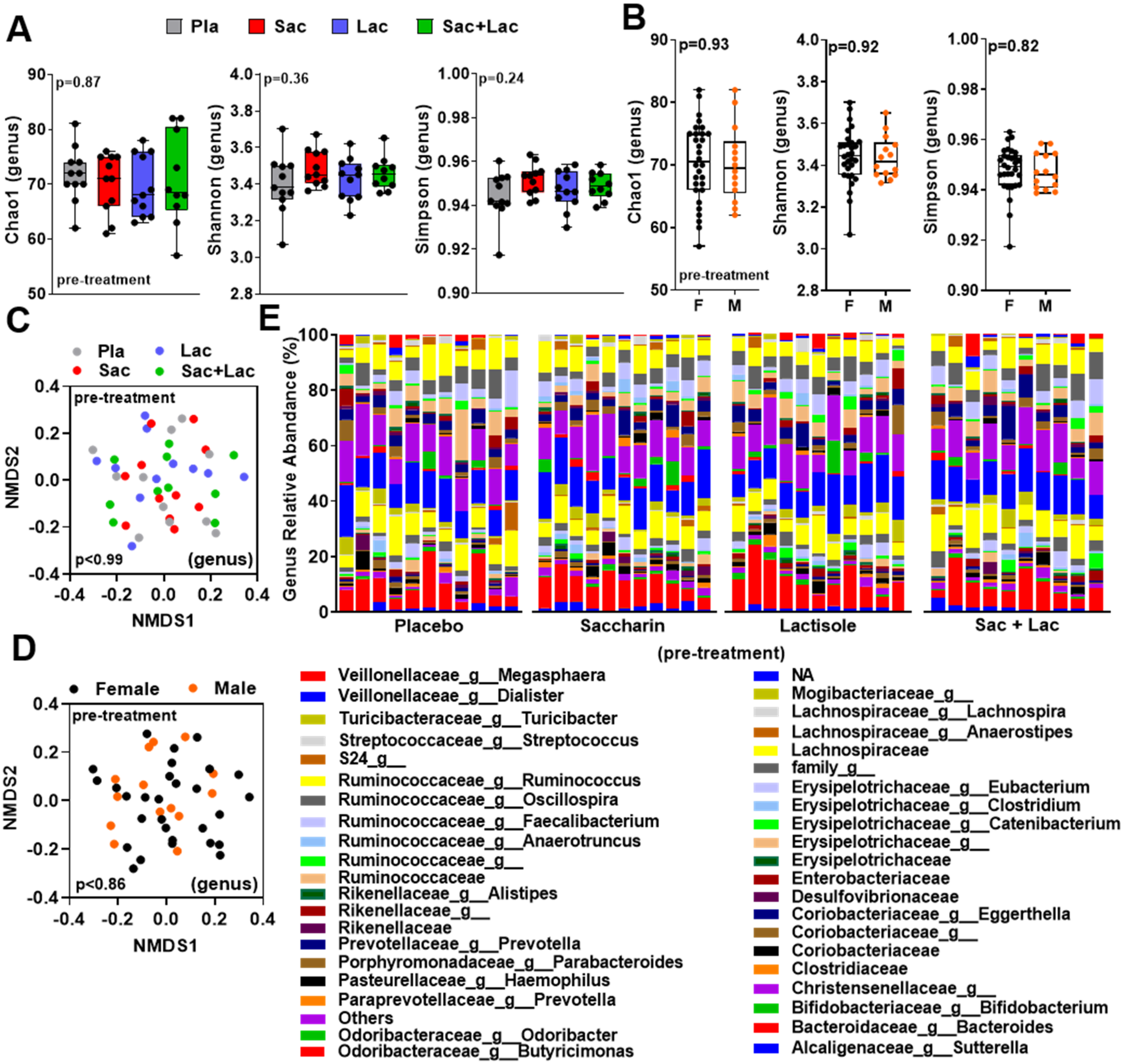

Supplemental figure.4 Pre-treatment comparisons of gut microbial diversity and composition (genus) in humans

(A) Alpha diversity indices (Chao1, Shannon, and Simpson) between treatment groups or **(B)** between genders at baseline (pre-treatment; detailed statistics, **Supp. Table.3**). **(C)** Nonmetric multidimensional scaling (NMDS) plots of Bray-Curtis dissimilarity between treatment groups or **(D)** between genders at baseline (pre-treatment; detailed statistics, **Supp. Table.4**). **(E)** Microbiota composition at the genus level of all randomized participants at baseline (pre-treatment; detailed statistics, **Supp. Table.5**). For (A), ANOVA, p-value. For (B), t-test p-value. For (C-D), PERMANOVA p-value. F, female; M, male.

Supp. Fig.5

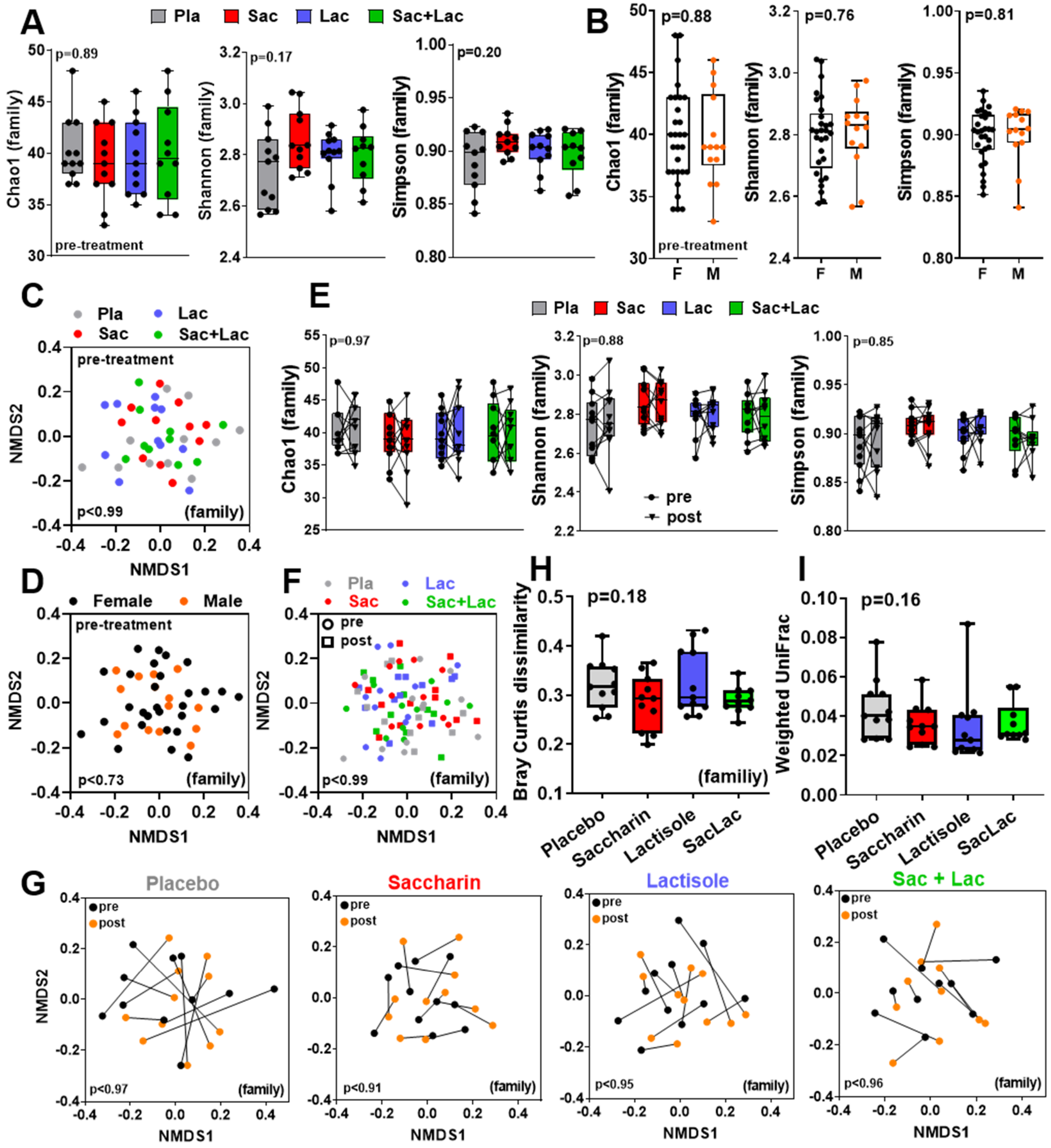

Supplemental figure.5 Pre-treatment comparisons and treatment effects on gut microbial diversity and composition in humans

(A) Alpha diversity indices (Chao1, Shannon, and Simpson) between treatment groups or **(B)** between genders at baseline (pre-treatment; detailed statistics, **Supp. Table.3**). **(C)** Nonmetric multidimensional scaling (NMDS) plots of Bray-Curtis dissimilarity between treatment groups or **(D)** between genders at baseline (pre-treatment; detailed statistics, **Supp. Table.4**). **(E)** Alpha diversity indices pre- and post-treatment (lines connect data from the same participant; detailed statistics, **Supp. Table.3**). **(F)** NMDS plots between all groups pre- and post-treatment, or **(G)** within each group (lines connect data from the same participant). **(H)** Within-subject Bray-Curtis dissimilarity (paired pre-post) for each treatment group (detailed statistics of beta diversity, **Supp. Table.4**). **(I)** Within-subject weighted UniFrac distance (paired pre-post) for each treatment group (detailed statistics, **Supp. Table.4**). For (A,H), ANOVA p-value. For (B), t-test p-value. For (C,D,F,G), PERMANOVA p-value. For (E), two-way ANOVA repeated measures, p-value for time x treatment effect. For (I), Kruskal-Wallis p-value. F, female; M, male.

Supp. Fig.6

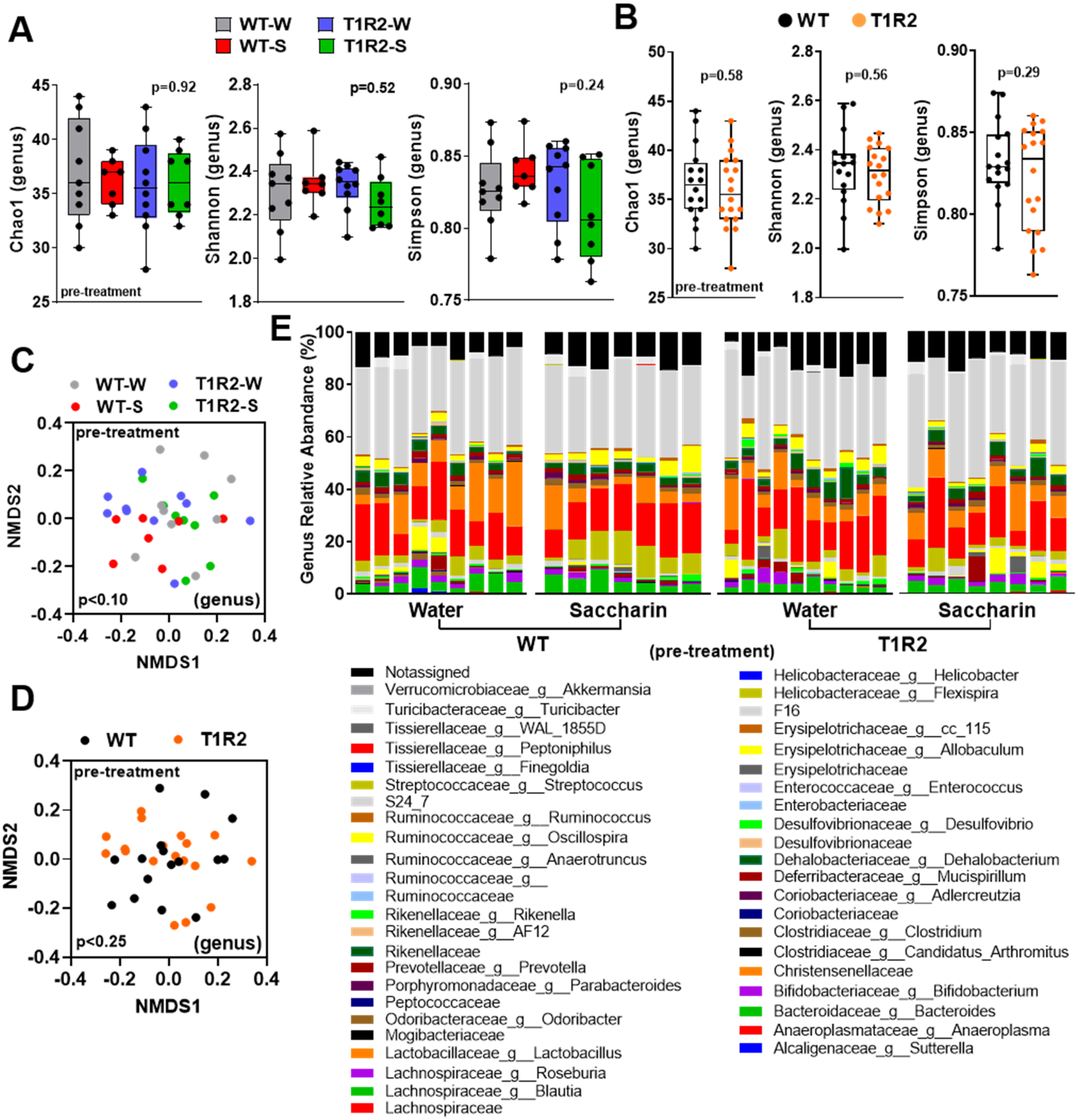

Supplemental figure.6 Pre-treatment comparisons of gut microbial diversity and composition (genus) in mice

(A) Alpha diversity indices (Chao1, Shannon, and Simpson) between treatment groups or **(B)** between genotypes at baseline (pre-treatment; detailed statistics, **Supp. Table.7**). **(C)** Nonmetric multidimensional scaling (NMDS) plots of Bray-Curtis dissimilarity between treatment groups or **(D)** between genotypes at baseline (pre-treatment; detailed statistics, **Supp. Table.8**). **(E)** Microbiota composition at the genus level of all mice at baseline (pre-treatment; detailed statistics, **Supp. Table.9**). For (A), ANOVA, p-value. For (B), t-test p-value. For (C-D), PERMANOVA p-value. W, water; S, saccharin.

Supp. Fig.7

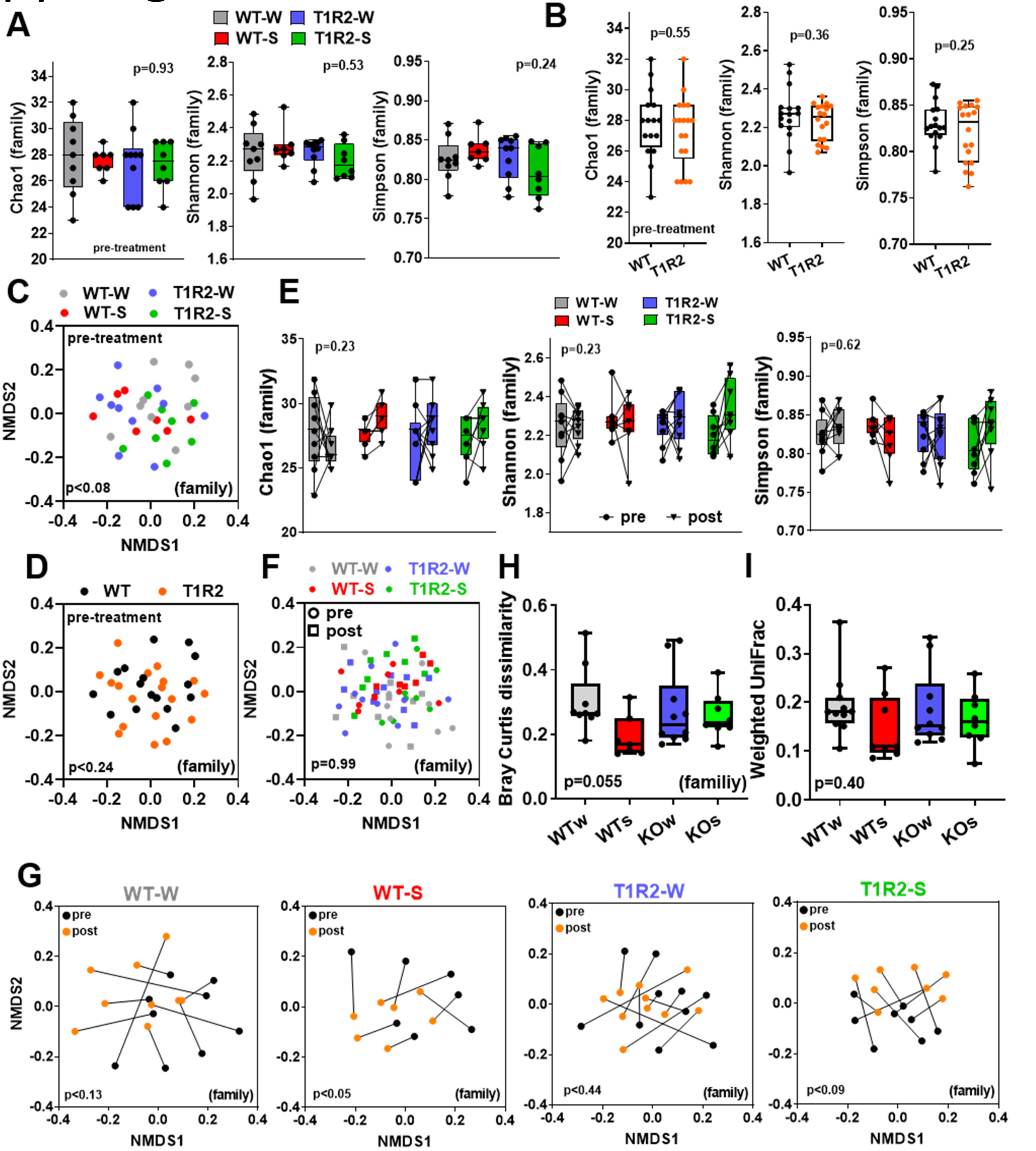

Supplemental figure.7 Pre-treatment comparisons and treatment effects on gut microbial diversity and composition in mice

(A) Alpha diversity indices (Chao1, Shannon, and Simpson) between treatment groups or **(B)** between genotypes at baseline (pre-treatment; detailed statistics, **Supp. Table.7**). **(C)** Nonmetric multidimensional scaling (NMDS) plots of Bray-Curtis dissimilarity between treatment groups or **(D)** between genotypes at baseline (pre-treatment; detailed statistics, **Supp. Table.8**). **(E)** Alpha diversity indices pre- and post-treatment (detailed statistics, **Supp. Table.7**). **(F)** NMDS plots between pre- and post-treatment of all groups, or **(G)** within each group. **(H)** Within-subject Bray-Curtis dissimilarity (paired pre-post) for each treatment (detailed statistics of beta diversity, **Supp. Table.8**). **(I)** Within-subject weighted UniFrac distance (paired pre-post) for each treatment group (detailed statistics, **Supp. Table.8**). For (A), ANOVA p-value. For (B), t-test p-value. For (C,D,F,G), PERMANOVA p-value. For (E), two-way ANOVA repeated measures, p-value for time x treatment effect. For (H,I), Kruskal-Wallis p-value.

Supp. Fig.8

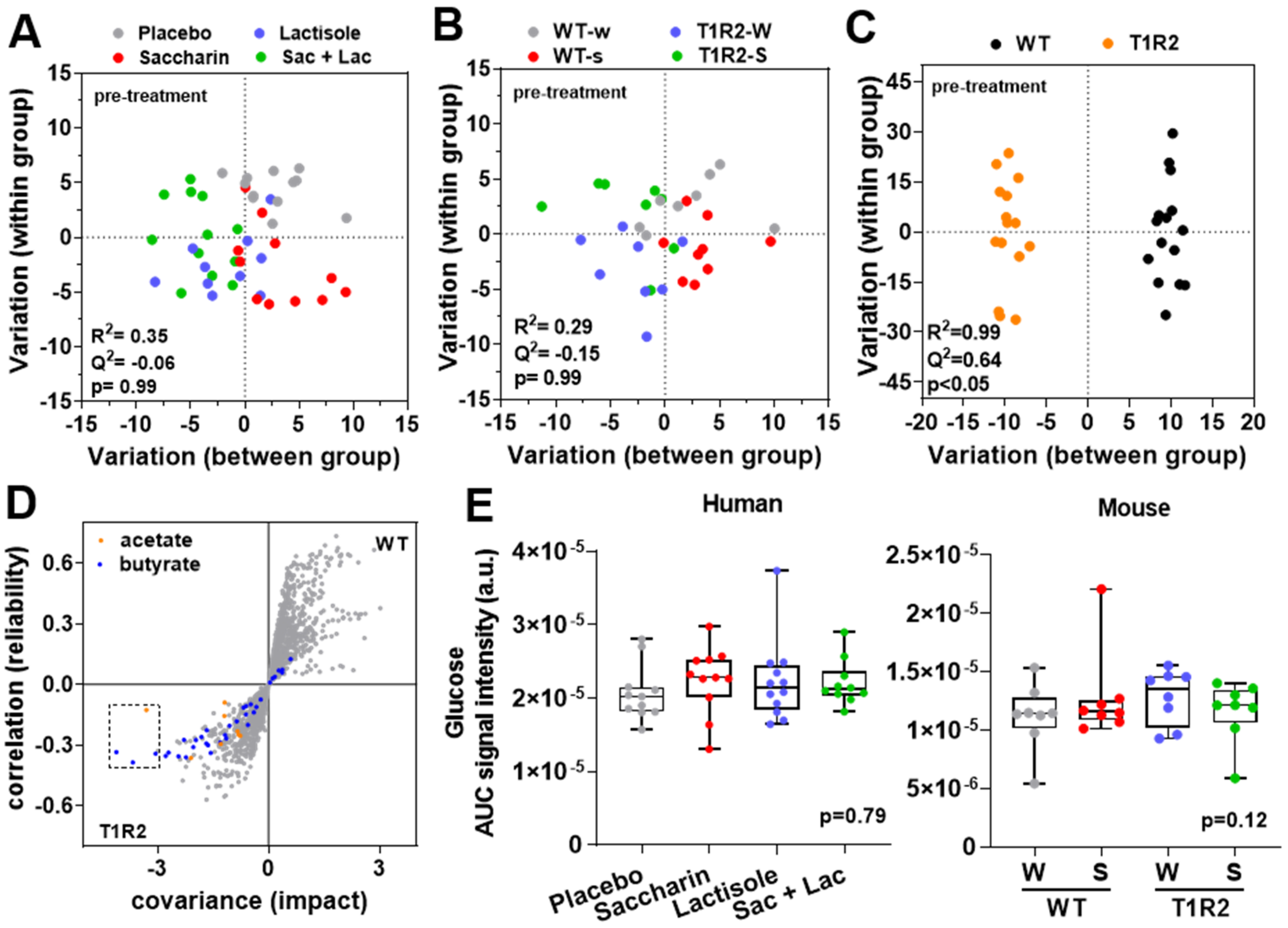

Supplemental figure.8 Pre-treatment fecal metabolomics in humans and mice

Pre-treatment variation in fecal metabolites using orthogonal partial least squares discriminant analyses (OPLS-DA) between treatment groups **(A)** in humans, **(B)** mice, or **(C)** between genotypes in mice. **(D)** Genotype metabolite distributions (S-plot) in pre-treatment fecal samples based on the OPLS-DA of NMR spectral bins (ppm). Rectangle includes statistically significant NMR spectral bins assigned to acetate and butyrate (increased in T1R2-KO samples). Orange and blue dots show all NMR spectral bins (ppm) assigned to acetate and butyrate, respectively (detailed statistics, **Supp. Table.11**). **(E)** Presence of glucose in human and mouse post-treatment fecal samples. For (A-C), R2; Q2; and CV-ANOVA p-value. For (E), ANOVA p-value (human), Two-way ANOVA p-value of genotype x treatment effect (mouse). W, water; S, saccharin.

Supp. Fig.9

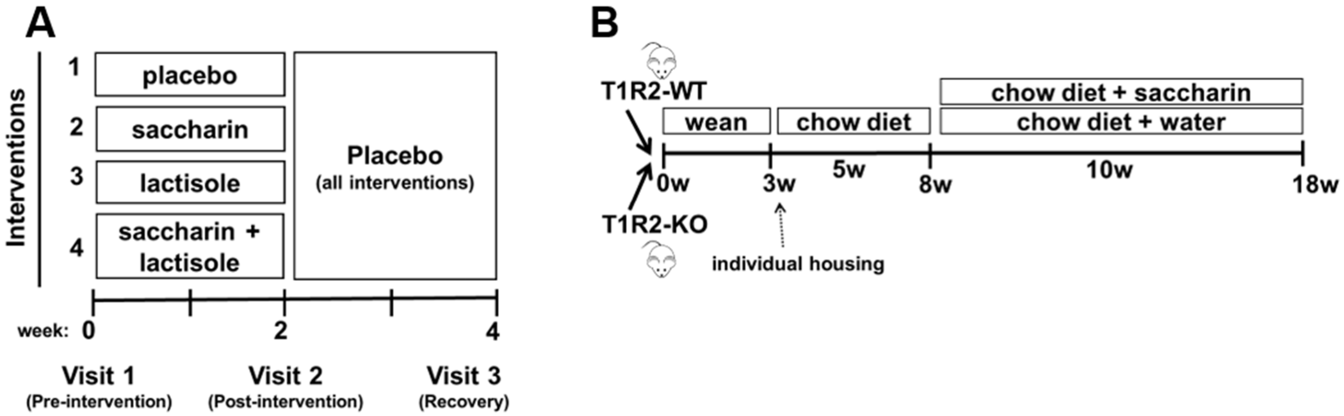

Supplemental figure.9 Experimental design of the human and mouse studies

Diagrams showing the experimental design of **(A)** human and **(B)** mouse studies.
